# Battery state of health estimation under fast charging via deep transfer learning

**DOI:** 10.1016/j.isci.2025.112235

**Published:** 2025-03-18

**Authors:** Jingyuan Zhao, Di Li, Yuqi Li, Dapai Shi, Jinrui Nan, Andrew F. Burke

**Affiliations:** 1 Institute of Transportation Studies, University of California, Davis, Davis, CA 95616, USA; 2 Hubei Longzhong Laboratory, Hubei University of Arts and Science, Xiangyang 441000, China; 3 Key Laboratory for Renewable Energy, Beijing Key Laboratory for New Energy Materials and Devices, Beijing National Laboratory for Condensed Matter Physics, Institute of Physics, Chinese Academy of Sciences, Beijing 100190, China; 4 Shenzhen Automotive Research Institute, Beijing Institute of Technology, Shenzhen 518000, China

**Keywords:** Electrochemical energy storage, Energy storage, Energy systems;

## Abstract

Accurate state of health (SOH) estimation is essential for effective lithium-ion battery management, particularly under fast-charging conditions with a constrained voltage window. This study proposes a hybrid deep neural network (DNN) learning model to improve SOH prediction. With approximately 22,000 parameters, the model effectively estimates battery capacity by combining local feature extraction (convolutional neural networks [CNNs]) and global dependency analysis (self-attention). The model was validated on 222 lithium iron phosphate (LFP) batteries, encompassing 146,074 cycles, with limited data availability in a state of charge (SOC) range of 80%–97%. Trained on fast-charging protocols (3.6C–8C charge, 4C discharge), it demonstrates high predictive accuracy, achieving a mean absolute percentage error (MAPE) of 3.89 mAh, a root-mean-square error (RMSE) of 4.79 mAh, and a coefficient of determination (R^2^) of 0.97. By integrating local and global analysis, this approach significantly enhances battery aging detection under fast-charging conditions, demonstrating strong potential for battery health management systems.

## Highlights

• Proposed a CNN and self-attention-based multi-fusion model for battery SOH estimation
• Two datasets of 222 LFP batteries are used for training, validation, and testing
• Achieves high accuracy using partial voltage and capacity in an end-to-end approach
• Transfer learning enhances generalization to capture cell-to-cell variations

## Graphical abstract

**Figure:**
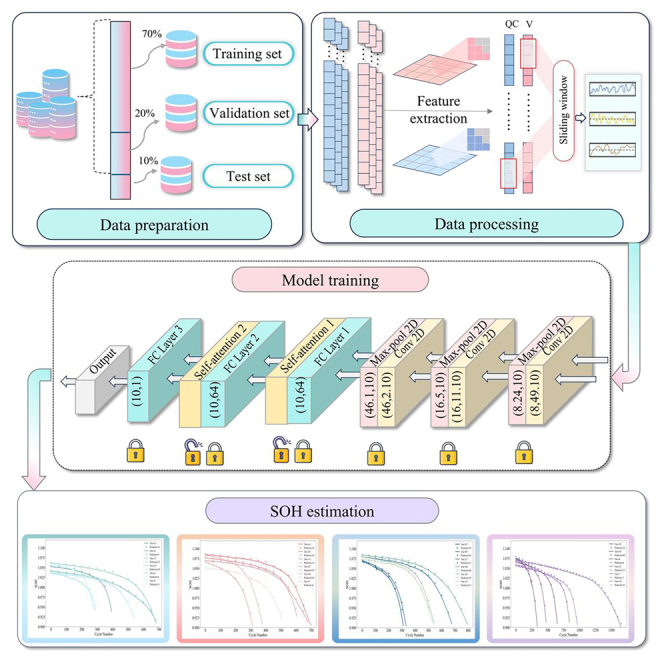

## Introduction

### Background

The global push for sustainable development and the transformation of energy systems have driven rapid advancements in new energy technologies, with lithium-ion batteries emerging as highly efficient energy storage solutions. These batteries are widely utilized across various applications, including electric vehicles, renewable energy storage systems, and consumer electronics.^1^^,^^2^ As governments worldwide implement stricter carbon emission regulations and promote green transportation and clean energy, the demand for lithium-ion batteries continues to grow. This surge in demand has spurred innovation, leading to significant improvements in battery technology.^3^ Batteries are now positioned as essential energy storage devices in the vehicle market, supporting both light-duty vehicles (LDVs)^4^ and medium- and heavy-duty vehicles (MHDVs).^5^ Enhanced energy density, technological advancements, and more mature supply chains have enabled batteries to meet the diverse performance and durability requirements of these sectors. Consequently, they play a pivotal role in driving the transition to electrified transportation, promoting sustainability and efficiency across the entire vehicle spectrum. However, in the transportation sector, the rapid development of electric vehicles has set higher standards for batteries.^6^ The performance of electric vehicles is closely related to the batteries they are equipped with, and the range, charging efficiency, and service life of the batteries directly affect the market competitiveness of the vehicles.^7^ Particularly as automakers continue to extend vehicle range, the capacity and energy density of battery packs must also increase. However, during long-term use and frequent charge-discharge cycles, the state of health (SOH) of batteries gradually declines, resulting in capacity degradation, increased internal resistance, and overall performance deterioration.^8^ This not only affects the driving range of the vehicle,^9^ but may also increase potential safety risks.^10^ Therefore, efficiently and accurately predicting SOH has become a major focus of research in both academia and industry.^11^

The aging process of lithium-ion batteries is complex and involves a variety of physical and chemical reactions. During each charge-discharge cycle, the insertion and extraction of lithium ions into and from the electrode materials cause changes in electrode structure, leading to material degradation.^12^^,^^13^ In addition, the decomposition of the electrolyte and the occurrence of side reactions can further accelerate battery aging. Over time, the capacity of battery gradually decreases, and its internal resistance increases, ultimately affecting energy output and power performance.^14^ In practical applications, the estimation of lithium-ion battery SOH faces multiple challenges. The complexity and variability of the battery aging process make estimation difficult, as battery performance degradation is influenced not only by internal materials but also by external factors such as the environment and usage conditions.^15^ In addition, SOH estimation requires real-time collection of battery operational data, including key parameters such as voltage, current, and temperature. The accuracy and frequency of these data collection are crucial to the precision of the estimations. These models must not only capture the nonlinear behavior of battery aging but also possess adaptability to account for changes in battery status over long-term use. Therefore, the design of SOH estimation models often involves knowledge from electrochemistry, materials science, and data analysis. With the rise of data-driven deep learning methods,^16^ and artificial intelligence technologies,^17^^,^^18^ an increasing number of data analysis tools and algorithms are being applied to battery SOH estimation, offering new solutions for intelligent battery management and efficient operations.^19^^,^^20^^,^^21^

### Literature review

In recent years, significant progress has been made in battery diagnostic technologies, with researchers and engineers proposing various methods to enhance estimation accuracy and real-world performance.^22^^,^^23^ Traditional SOH estimation methods often rely on electrochemical models based on physical mechanisms, such as equivalent circuit models and extended electrochemical models. These models can accurately simulate the internal behavior of batteries, but due to the complexity of battery aging processes and individual variability, they face many limitations in practical applications.^24^^,^^25^^,^^26^^,^^27^ SOH estimation methods based on physical models typically require precise battery parameters and complex experimental measurement equipment, making them difficult to apply broadly in large-scale and diverse application scenarios.^28^^,^^29^ In addition, physical models struggle to adapt to different types of batteries and operating conditions. In response to these challenges, researchers have gradually shifted toward data-driven methods to achieve more generalized and efficient SOH estimation.^30^^,^^31^^,^^32^^,^^33^ These methods automatically extract features from the data and make estimations by collecting historical battery operation data (such as voltage, current, temperature, etc.) and using machine learning and deep learning techniques, no longer relying on complex experimental parameters and physical mechanism models.^34^^,^^35^^,^^36^ Classic machine learning methods, such as support vector machines,^37^^,^^38^ random forests and Gaussian process regression,^39^ are widely used for battery SOH estimation. These methods build nonlinear estimation models by extracting manually designed features from battery operational data, thus estimating the battery SOH.^40^^,^^41^ However, machine learning methods are heavily dependent on features, and manually designed features struggle to capture deep-level information in the aging process of batteries when dealing with complex time-series data.

The rise of deep learning has brought new opportunities for battery SOH estimation. Deep learning models have become powerful tools in battery diagnosis and performance estimation due to their ability to process large-scale complex datasets, reveal intricate patterns, and provide high estimation accuracy.^42^^,^^43^^,^^44^ Deep learning models can automatically learn and extract features from data, showing significant advantages, especially when dealing with complex time-series data.^45^^,^^46^ Convolutional neural networks (CNNs) excel at extracting local features from the spatial dimensions of battery data,^47^ while long short-term memory (LSTM) can capture long-term dependencies in time series.^48^ For instance, one study used LSTM networks and Pearson correlation analysis to identify relevant features and model the battery degradation process, achieving high accuracy and low estimation error in SOH estimation by using real-world battery data from electric buses.^49^ Another study proposed a CNN-based approach to simultaneously estimate the health and charge states of a battery using only a short period of charging data, overcoming the limitation of traditional studies that rely only on specific operational data to estimate a single state.^50^ Building on the integration of physical and data-driven methods, a different study combines physical information with a deep learning framework, demonstrating its capability to provide accurate SOH estimates even under varying operating conditions, which highlights the flexibility of such methods in dynamic environments.^51^ Furthermore, to address the challenge of lacking target battery labels, a study developed a deep learning framework capable of accurately estimating the SOH without the need for traditional, time-consuming, and resource-intensive degradation experiments, showcasing the promise of rapid development of battery management algorithms.^52^ However, the training of deep learning models usually requires a large amount of labeled data, which poses a greater challenge in practical applications, and limits the generalization ability of the models, especially when dealing with diverse battery types and complex working conditions. As a result, researchers have begun exploring the application of transfer learning in battery SOH estimation.^53^ Transfer learning enhances the generalization ability of the model by pre-training on one domain or dataset and then fine-tuning it for application in another domain, thus reducing reliance on large-scale labeled data. For example, one study combined LSTM networks with transfer learning strategies, achieving accurate SOH estimation using only a small amount of data.^54^ Similarly, a study proposed a method for predicting battery SOH through transfer learning and online model correction.^55^ This research designed a self-correction strategy to retrain the regression model, gradually achieving optimal estimation performance during the operation cycle. Another study introduced a Bayesian transfer learning framework that captures the heterogeneity of battery degradation using a mixed-effects model. By updating the parameters with Bayes theorem, this framework facilitates SOH estimation across various application scenarios.^56^ In addition, a study utilizes temporal migration learning to characterize the nonlinear aging trajectories of batteries through unsupervised segmentation and adaptively adjusts feature alignment for accurate battery health prediction.^57^ Recently, our team demonstrated that the multi-fusion model is a powerful tool for evaluating capacity in nickel cobalt aluminum (NCA) and nickel cobalt manganese (NCM) cells using transfer learning.^58^ The results highlight its ability to reduce computational complexity, energy consumption, and memory usage while maintaining high accuracy and robust generalization capabilities.

Hybrid models in battery SOH estimation are becoming a key approach for tackling complex estimation tasks. By integrating different types of models, these methods can handle the diverse characteristics of battery data, improving both estimation accuracy and reliability.^59^ These models combine various deep learning architectures or traditional machine learning methods, leveraging the strengths of each to enhance estimation precision and model robustness.^60^^,^^61^^,^^62^ Compared to single models, hybrid models are more effective at processing complex battery data, particularly excelling in handling multimodal and time-series data. For instance, one study proposed an indirect hybrid model for online battery estimation and health management.^63^ This model extracts two aging features from partial charging data to quantitatively evaluate battery health. Another study explored an interpretable hybrid framework that not only provides precise point estimation but also quantifies uncertainty in SOH predictions, improving the reliability of battery health assessment.^64^ To further improve the estimation accuracy, another study integrates CNN and transformer models to solve the battery pack inconsistency problem using hierarchical feature extraction and gray correlation analysis, which effectively improves the SOH estimation accuracy.^65^ Furthermore, considering the effect of ambient noise, another study developed a framework combining denoising CNNs with a CNN, where the denoising module mitigates the ambient noise and thus improves the robustness of SOH evaluation.^66^ In our recent work, we developed a predictive pre-trained transformer (PPT) model with 1.87 million parameters to improve battery health diagnosis by capturing both short- and long-term patterns in time-series data.^67^ By leveraging partial charge data and transfer learning, the model achieved high diagnostic accuracy, reduced computational costs, and demonstrated strong generalization capabilities, making it an effective tool for real-time battery health and lifetime assessments. This advancement highlights the transformative potential of integrating large-scale transformer neural networks with transfer learning in battery state estimation.^68^ These methods have significantly improved model generalization, reduced reliance on extensive datasets, and enhanced estimation accuracy, paving the way for more efficient and scalable diagnostic solutions.

While current methods demonstrate strong predictive performance and flexibility, significant challenges persist. Although the application of transformer and large-scale pre-trained models have demonstrated strong feature extraction and prediction capabilities in recent studies, they consume high computational resources and are still insufficiently interpretable in battery SOH estimation. On the other hand, most of the current deep learning models rely on data trained under specific operating conditions and are difficult to maintain high estimation accuracies under different charging and discharging rates, temperatures, and load conditions. Although existing research has proposed some adaptive methods, such as strategies based on migration learning and Bayesian frameworks, the stability and adaptability of the models are still insufficient when facing extreme operating conditions. Ensuring model robustness across diverse operating conditions, such as fast charging rates ranging from 3C to 8C, and improving real-time performance in practical applications remain critical areas for improvement. As innovative algorithms continue to emerge and battery big data grows, future research should prioritize the development of versatile hybrid models. These models must enhance generalization, computational efficiency, and resource optimization to address the increasingly complex demands of battery health management effectively.

### Structure and contribution

Accurate estimation of battery SOH has long been a complex challenge, primarily due to the degradation process of batteries being influenced by various factors, including operational conditions, usage patterns, and environmental influences. To address this issue, this study proposes a deep learning model that integrates CNN with a multi-head self-attention mechanism. By combining a two-dimensional CNN with the multi-head self-attention mechanism, the model effectively extracts deep features from the voltage-capacity curves, capturing the intricate interdependencies within the data, thereby significantly enhancing overall predictive performance. Specifically, the CNN excels at extracting local features from time-series data, while the self-attention mechanism improves estimation accuracy by capturing global dependencies between different time steps.

With the introduction of large-scale datasets, the complexity faced by the model increases, and traditional methods often struggle to capture long-term dependencies effectively, leading to overfitting or underfitting issues. To overcome these challenges, the proposed model offers several key contributions. First, a systematic data preprocessing pipeline was developed, including data interpolation, differential feature extraction, and the sliding window technique. This process ensures high-quality input data, effectively capturing the fundamental trends of battery aging and performance degradation, thereby significantly improving the efficacy of model training, as well as the accuracy and reliability of estimations. Second, the self-attention mechanism enhances the ability of the model to capture long-range dependencies, facilitating the identification of subtle trends in battery health changes. Moreover, by employing transfer learning techniques to fine-tune the model, we increase its generalization ability while reducing the risk of overfitting, maintaining high predictive accuracy. Furthermore, the model efficiently processes large-scale battery cycle data, demonstrating robustness and adaptability, particularly in SOH estimation for lithium iron phosphate (LFP) batteries. This underscores the potential of the model for broad application in battery health management systems.

This study is divided into four main sections. The second part introduces the experimental data and preprocessing workflow; The third part details the theoretical foundation and specific architecture of the model; The fourth part provides a multi-faceted discussion and analysis of the estimation results and performance of the model, while the final part concludes with a summary of the key contributions and findings of the study.

### Experimental data

#### Data overview

In this study, the dataset was generated from data collected from commercial (LFP)/graphite batteries in two different experiments. The batteries were tested in a temperature-controlled environment (30°C), with the discharge process conducted under the same constant current-constant voltage (CC-CV) mode, discharging to 2.0 V. The lifetime of the battery was defined as the number of cycles until the discharge capacity dropped to 80% of the nominal capacity. Key parameters, including voltage, current, internal resistance, and temperature, were recorded during both the charging and discharging processes across both datasets, providing comprehensive feature inputs for model training and estimation. Each experiment assessed the battery cycle life under various charging rates and strategies, offering a broad combination of variables for the dataset. The first dataset consists of data from 177 batteries,^69^ which were cycled under 72 different one-step and two-step fast charging strategies, producing 111,211 cycles. The charging rates ranged from 3.6C to 6C, with charging times varying from 9 to 13.3 min. The second dataset was generated through experiments using 45 six-step fast charging protocols,^70^ each with a uniform charging protocol, resulting in a total of 37,955 cycles. These comprehensive datasets capture battery performance under fast charging conditions and incorporate diverse charging schemes to support model training and validation across a wide range of operational scenarios, thereby enhancing the generalization capability and estimation accuracy of the deep learning model.

**Figure 1:**
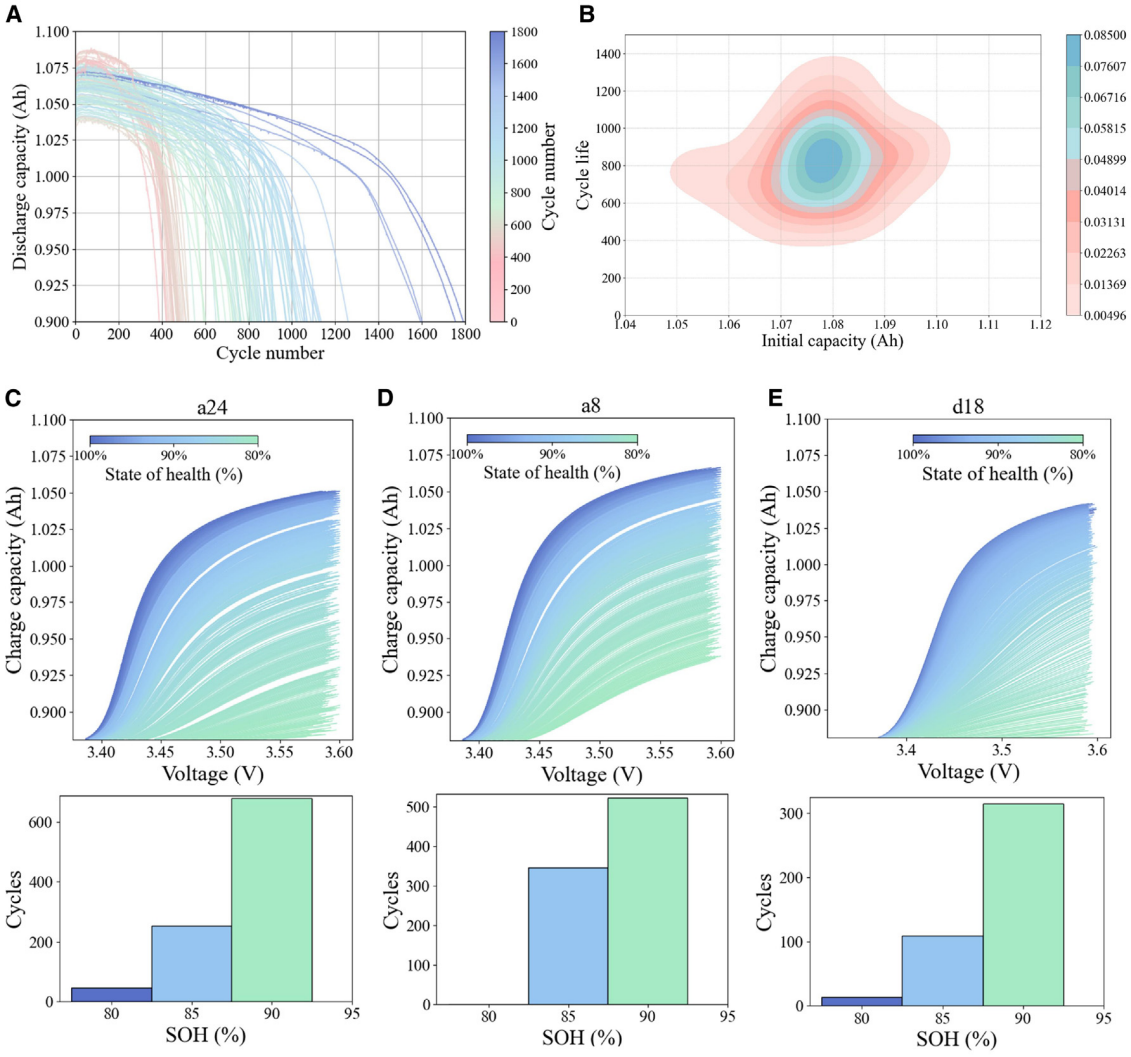
Data group diagram This figure explores the relationship between battery degradation, cycle life, and SOH. (A) Visualizes the discharge capacity over cycle numbers, showing variations across batteries. (B) Displays the correlation between initial capacity and cycle life. (C–E) Respectively illustrate the relationship between charge capacity and voltage under different health states, while the bar charts depict the number of cycles corresponding to varying health percentages for batteries a24, a8, and d18. These analyses provide insights into the lifespan and performance of the batteries under various operating conditions.

The proposed experiment utilized the entire dataset from the first study and a portion of the dataset from the second study, comprising a total of 222 batteries, generating 146,074 cycles (Table 1). Figure 1A illustrates the relationship between discharge capacity and the number of cycles over a span of 1,800 cycles, with the color representing cycle life. The results indicate that, for most cells, discharge capacity declines rapidly between 300 and 1,200 cycles. The initial capacities of most cells are concentrated between 1.06 and 1.08 Ah, corresponding to a cycle life of approximately 800–1,200 cycles. Instances where the cycle life is less than 500 or exceeds 1,500 are relatively rare (Figure 1B). To further analyze the charging capacity behavior of batteries at different voltage levels, all batteries were labeled, with the batteries from the first dataset divided into three subsets marked as a, b, c, and d, and the batteries from the second dataset marked as e. Three batteries (a24, a8, and d18) were randomly selected for detailed illustration. In Figures 1A, 1E, and 1D, the color gradient from deep blue to light green represents the transition of batteries from a new state (100% SOH) to a degraded state (80% SOH). Below the data of each battery, bar charts display the number of cycles corresponding to different SOH levels.

**Table 1:** Overview of the dataset used in this study

Dataset	Battery information	Charge-discharge strategies/number of charge-discharge strategies	Temperature	Degradation range
#1	Battery count: 177; Nominal voltage: 3.3 V; nominal capacity: 1.1 Ah; cutoff voltage: 2.0 V; compositions: LFP/graphite	Charging: 0–80% state of charge (SOC) at 3.6C–6C; CC charging to cutoff voltage; CV charging with cutoff current C/50; discharging: 100%–0% SOC at 4C to 2.0 V.	30°C	100%–80% SOH
#2	Battery count: 45; nominal voltage: 3.3 V; nominal capacity: 1.1 Ah; cutoff voltage: 2.0 V; compositions: LFP/graphite	Charging: multi-step strategy with rates from 3.6C to 8C; SOC 0%–80%; discharging: 100%–0% SOC at 4C to 2.0 V.	30°C	100%–80% SOH

#### Data preprocessing

The data preprocessing phase involves several key steps, including raw data extraction, feature normalization, feature construction, and dataset segmentation. The datasets record battery charge and discharge cycles, generating time-series data containing information on cycle life, charge capacity, discharge capacity, voltage, and temperature, which are used to construct a training dataset for model learning. During the data preparation process, invalid battery data were first removed, including noisy channels and data from 20 batteries that did not reach 80% discharge capacity. Then, battery data with more than 200 cycles were selected. Next, the extracted key features were normalized, and sliding windows were applied to generate the input features for the model. Finally, the preprocessed data were split into training, validation, and test sets, with random shuffling applied to ensure unbiased distribution. This complete preprocessing pipeline ensures data integrity, standardization, and usability, thus enhancing the efficiency and accuracy of subsequent model training and validation.

#### Feature extraction and normalization

Feature extraction plays a pivotal role in data preprocessing, as it seeks to isolate critical information from the raw battery cycle data that indicates battery performance and health. In this study, datasets from five distinct batches of batteries were utilized, with voltage and charge capacity chosen as the main features. Discharge capacity was also included as an auxiliary label to enhance the understanding of degradation patterns of batteries. To capture the performance decay as the cycle count increases, the voltage curve of each cycle was compared with that of the 10th cycle, calculating the difference in voltage and the difference in charge capacity as derived features. These features were interpolated to a fixed length of 100 time steps using a linear interpolation function to ensure data continuity and consistency. The resulting feature matrix contains voltage, charge capacity, voltage difference, and charge capacity difference for each cycle, which serve as input features for the model.

To ensure the model efficiently handles the input data, all extracted features were normalized. The goal of normalization is to scale features of different magnitudes into the same numerical range, eliminating biases and improving the performance of model training. Voltage features were normalized within the range of $v_{low}$ = 3.36 to $v_{upp}$ = 3.60, while charge capacity features were scaled between $q_{low}$ = 0.61 and $q_{upp}$ = 1.19. The differences in voltage and charge capacity were also normalized to ensure consistency across all features. Normalization was performed by subtracting the minimum value and dividing by the range, ensuring linear transformation. This process enables faster model convergence and reduces numerical instability during gradient descent.

#### Sliding window feature construction

To capture the trends in the behavior of batteries across multiple charge and discharge cycles, a sliding window mechanism was employed for feature construction. The sliding window length was set to 100 time steps, with a stride of 1 cycle. For the cycle data of each battery, the normalized feature matrix (containing voltage, charge capacity, voltage difference, and charge capacity difference) was sliced into tensors with a shape of (100, 100, 4). This windowed feature construction effectively captures trends in battery behavior over multiple cycles, significantly enhancing the ability of the model to detect battery life trends and improving its generalization performance.

#### Dataset segmentation

This study analyzes two datasets comprising hundreds of distinct charging protocols. Each cell operates under a unique protocol, representing various operational conditions to assess their impact on battery degradation and aging. The first dataset follows a four-step fast-charging protocol with rates ranging from 3.6C to 6C. In contrast, the second dataset uses a more complex six-step protocol, with charging rates ranging from 3.6C to 8C. This method introduces varying stress levels at different stages, providing a detailed understanding of how multi-stage charging influences battery aging. By analyzing health and aging estimation for each cell, the study highlights the diverse aging trajectories driven by different charging rates.

**Figure 2:**
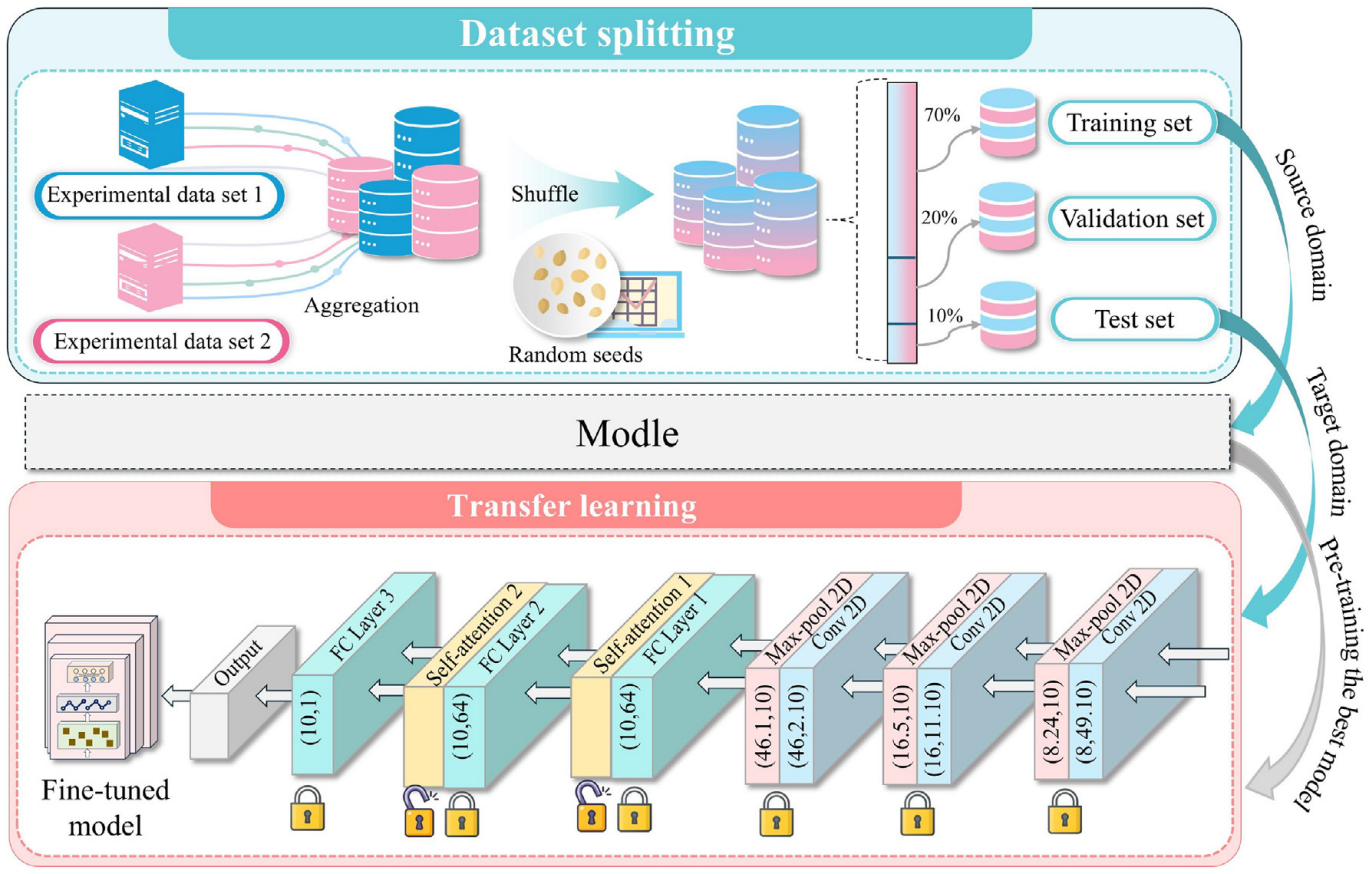
Dataset splitting process and transfer learning model architecture The upper section depicts the aggregation of two experimental datasets. The dataset is partitioned into training, validation, and test subsets in a ratio of 70:20:10. Pre-training is performed on the source domain, and the target domain is utilized for fine-tuning the model. The lower section demonstrates the transfer learning process, where the model undergoes pre-training using convolutional and self-attention layers, followed by fine-tuning. Specific layers are locked to preserve learned features, enabling the optimization of performance for the target domain.

To ensure robust model training and evaluation, the two datasets were initially merged and subsequently divided into training, validation, and test sets. The distribution was set at 70% for training, 10% for validation, and 20% for testing. This specific partitioning process is detailed in Figure 2. Each segment contains the complete set of features for its respective batteries: 70% of the batteries form the training set for optimizing model parameters; 10% comprise the validation set for hyperparameter tuning; and the remaining 20% constitute the test set for evaluating the generalization performance. This structured approach ensures that each phase of model development is supported by appropriate data, facilitating accurate and effective analysis.

#### Model architecture

To predict health status, a nonlinear mapping between the extracted features and capacity degradation needs to be established. To achieve this, we designed a model that combines two powerful feature extraction techniques: CNN and the self-attention mechanism. The convolutional layers capture local variations in battery features, while the self-attention mechanism identifies relationships across different time steps at a global level, effectively enhancing estimation accuracy.

The model initially applies a series of convolutional layers to extract input features, introducing nonlinearity through activation functions. Each convolutional layer is paired with a max-pooling layer, which reduces dimensionality while retaining essential information in the feature space. The feature maps generated by the convolutional layers are subsequently compressed and processed through fully connected layers, with activation functions enhancing nonlinearity and minimizing information loss. Finally, after traversing two fully connected layers, the model utilizes a multi-head self-attention mechanism to reinforce feature relationships, capturing global dependencies and improving the effectiveness of feature representation.

#### Convolutional layer design

**Figure 3:**
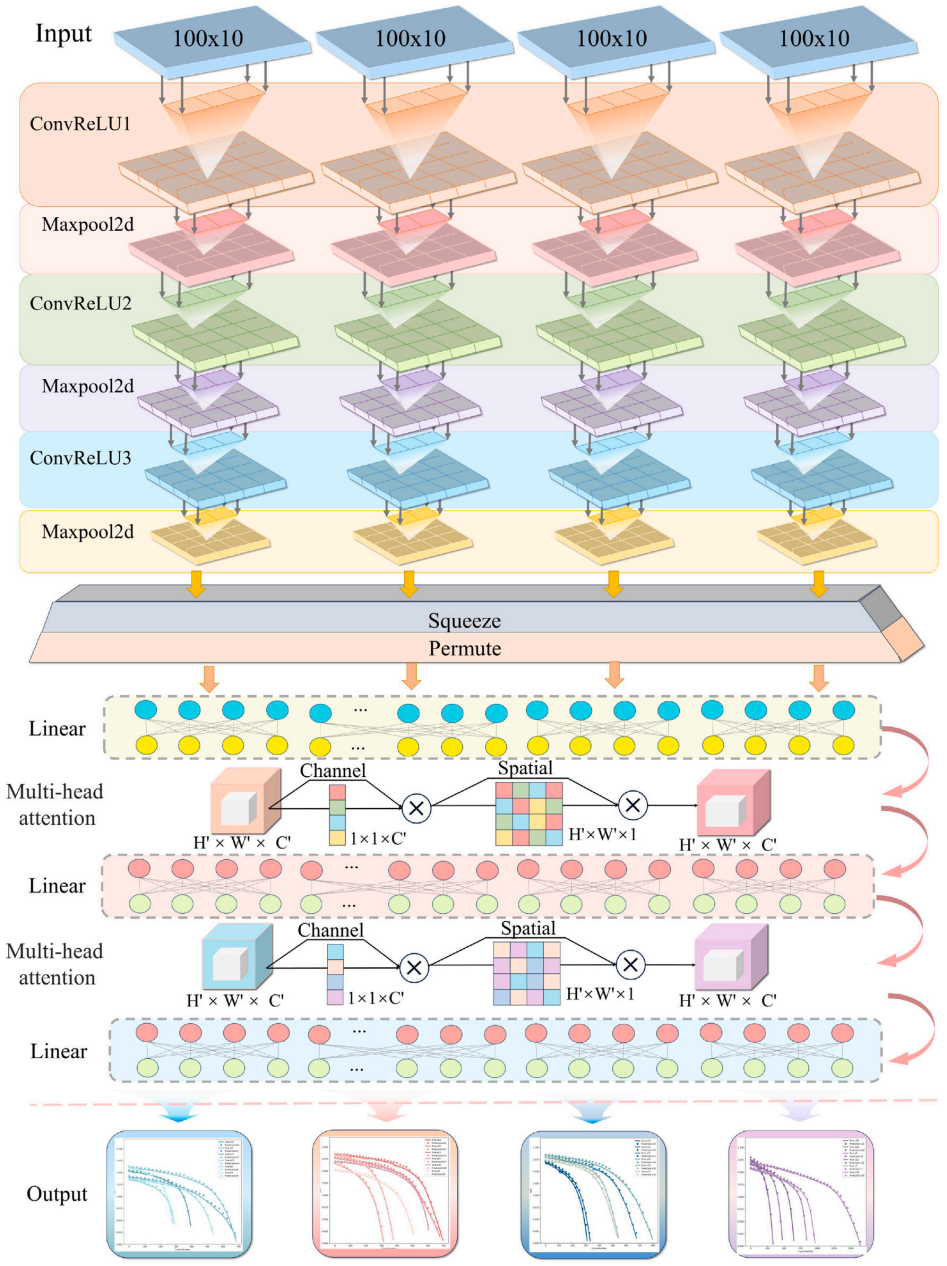
Overview of the proposed SOH estimation model framework This figure illustrates a hybrid architecture that integrates convolutional layers and multi-head attention mechanisms for battery health estimation. The model processes input data through three convolutional layers, each followed by max-pooling for feature extraction and dimensionality reduction. The features are then passed through a squeeze-permute operation and further refined using multi-head attention layers, which apply both channel and spatial attention. The final output generates estimations related to battery performance over time, enabling accurate SOH estimation under varying conditions.

The first part of the model consists of three convolutional layers designed to extract local temporal features from the input data. By progressively capturing high-dimensional characteristics of the input signals, this design enables the model to effectively capture the dynamic variations in battery SOH. As shown in Figure 3, the configuration of each convolutional block is defined by the convolutional rectified linear unit (RELU) module, where each convolutional layer includes convolution, activation, and optionally batch normalization operations. The first convolutional layer processes data with four input channels and outputs eight channels. The kernel size is set to 3 × 1 with a stride of 2 × 1, which reduces the spatial dimensions while preserving local dependencies within the data. Subsequently, a MaxPool2d layer further reduces the feature map size.

Convolutional blocks 2 and 3 continue this progressive feature extraction pattern, expanding the number of channels to 16 and 64 (Table 2), respectively. Each convolution is followed by a max pooling operation. After each convolutional layer, a RELU activation function is applied to introduce nonlinearity into the model, enabling it to capture complex patterns in the data and ensuring efficient expression of nonlinear features. In addition, the max pooling operation reduces redundant information in the feature maps, lowering computational cost. Through these three convolutional and pooling layers, the model gradually extracts deep features from the initial input, laying a solid foundation for the subsequent self-attention mechanism for SOH estimation.

**Table 2:** Model network structural hyperparameters

Parameter	Value	Explanation
n_cyc	30	Number of previous cycles used for model input.
batch_size	512	Batch size for training and validation.
Lr	8e-4	Learning rate for training.
num_epochs	12,000	Maximum number of training epochs.
Patience	1,600	Early stopping patience.
Alpha	[0.1] ∗ 10	Capacity loss weight during pre-training.
in_ch	4	Number of input channels for convolution layers.
out_ch	[8, 16, 64]	Number of output channels for convolution layers.
Kernel	3	Kernel size for convolution layers.
Stride	2	Stride for convolution layers.
Padding	0	Padding for convolution layers.
embed_dim	64	Embedding dimension for multi-head attention layers.
num_heads	2	Number of attention heads in multi-head attention layers.
Dropout	0.3	Dropout rate for multi-head attention layers.
dense_1	64	Number of neurons in the first dense layer.
dense_2	64	Number of neurons in the second dense layer.
finetune_lr	2e-5	Learning rate for fine-tuning.
train_alpha	[0.09] ∗ 9 + [0]	Fine-tuning capacity loss weights during training.
valid_alpha	[0.09] ∗9 + [0]	Fine-tuning capacity loss weights during validation.
finetune_epochs	800	Maximum number of fine-tuning epochs.

#### Self-attention mechanism

Building on the high-dimensional feature maps extracted by the convolutional layers, the model further captures the long-range dependencies over time during the charge-discharge cycles of the battery using a self-attention mechanism. The model includes two multi-head self-attention modules, each consisting of two attention heads and an embedding dimension of 64 (Table 2). The core concept of this component is that, through the multi-head attention mechanism, the model can simultaneously focus on multiple time steps within the input data, enabling more accurate detection of the changing trends in the battery SOH.

**Figure 4:**
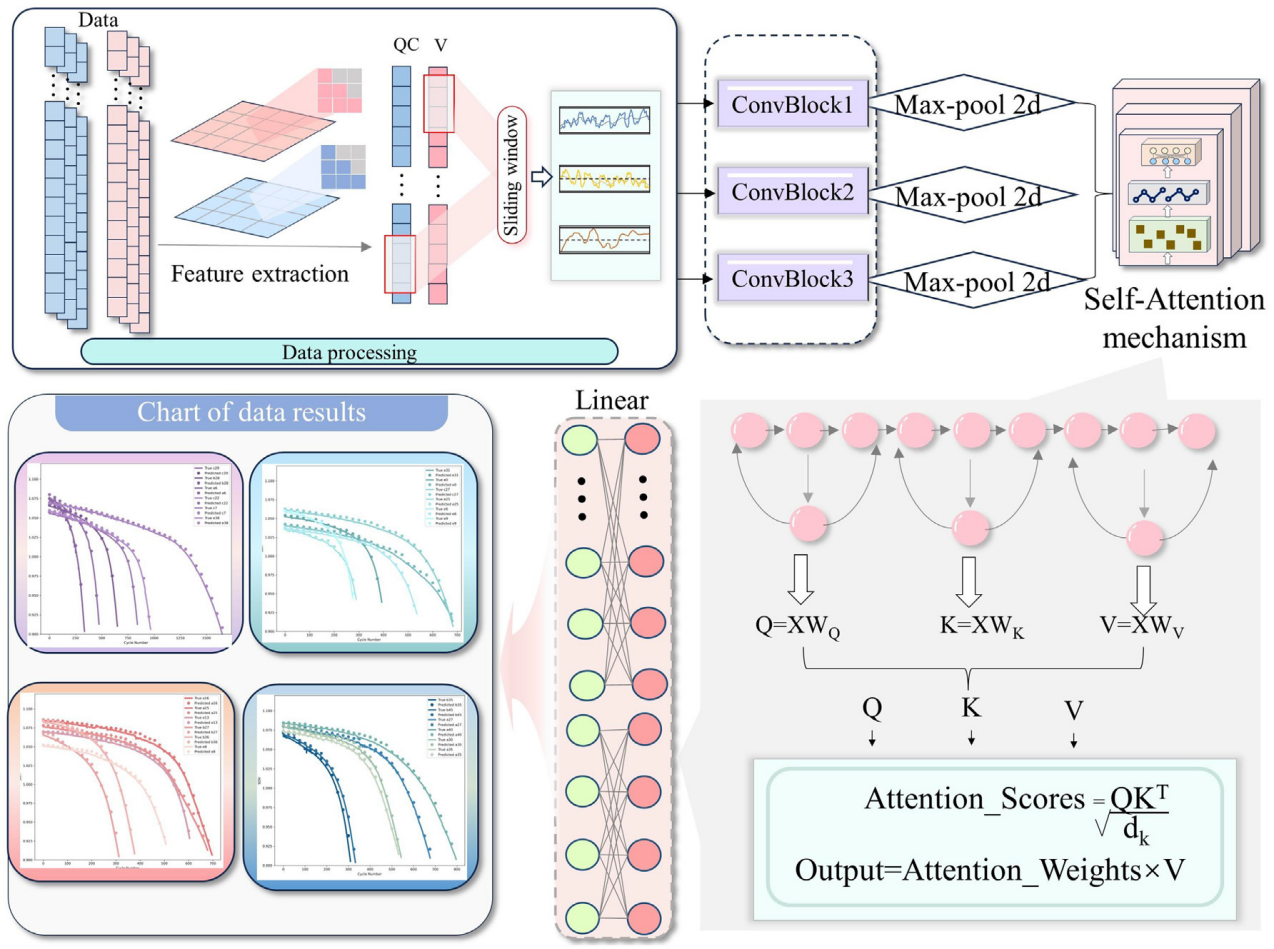
Model architecture diagram This figure demonstrates a process that combines feature extraction and self-attention mechanisms for battery health estimation. The input data undergoes processing through sliding windows and feature extraction steps before being passed into three convolutional blocks, each followed by max-pooling. The processed features are further refined using a self-attention mechanism, where queries, keys, and values are calculated to generate attention scores. The final output presents charts that visualize the estimation results for different battery conditions, improving the accuracy of SOH estimation.

The output from the convolutional layers is initially processed through convolution operations, with the RELU activation function applied to introduce nonlinearity. The resulting feature maps are then reduced in size using max-pooling layers to lower their dimensionality. For input data of shape [B, 4, 100, 10]—where B denotes the batch size, 4 represents the input feature dimension, 100 is the time step length, and 10 corresponds to the sequence length—the extracted features are subsequently passed to the self-attention layer (Table 3). The specific model hyperparameter settings are detailed in Table 2. Figure 4 provides a detailed representation of the operational flow of the self-attention mechanism, illustrating how attention is distributed across channel and spatial dimensions. By utilizing global contextual information, the model can capture both broad trends and subtle variations in the cell operational characteristics, significantly improving accuracy.

**Table 3:** Model network structural parameters

Index	Layer	Weight parameters	Trainable parameters
1	Conv2d (4, 8, kernel_size=(3, 1), stride=(2, 1))	4 ∗ 8 ∗ 3 ∗ 1 = 96	96
2	Conv2d (8, 16, kernel_size=(3, 1), stride=(2, 1))	8 ∗ 16 ∗ 3 ∗ 1 = 384	384
3	Conv2d (16, 64, kernel_size=(3, 1), stride=(2, 1))	16 ∗ 64 ∗ 3 ∗ 1 = 3,072	3,072
4	Linear (in_features = 64, out_features = 64)	64 ∗ 64 = 4,096	4,096
5	MultiheadAttention (out_proj = 64, 64)	64 ∗ 64 = 4,096	4,096
6	Linear (in_features = 64, out_features = 64)	64 ∗ 64 = 4,096	4,096
7	MultiheadAttention (out_proj = 64, 64)	64 ∗ 64 = 4,096	4,096
8	Linear (dense_soh: in_features = 64, out_features = 1)	64 ∗ 1 = 64	64

The self-attention mechanism operates by calculating the attention weights between different features using query (Q), key (K), and value (V) vectors. The attention mechanism is computed as follows:

(1) $$A\text{ttention}\left(Q,K,V\right)=\text{softmax}\left(\frac{QK^{T}}{\sqrt{d_{k}}}\right)V$$

where, Q, K, and V represent the query, key, and value matrices, respectively. By applying this attention mechanism, the model can assign different weights to various time steps, allowing it to focus on the most relevant periods within the charge-discharge cycles of the battery. This combination of convolutional layers for local feature extraction and self-attention mechanisms for capturing global dependencies enables the model to effectively learn the behavior of the battery over time, thereby improving the accuracy.

#### Full connectivity layer and output design

Once the self-attention mechanism has captured the global dependencies within the input features, the model further processes these features through fully connected layers to produce the final SOH estimation. Initially, the model transforms the 64-dimensional feature maps into a more detailed feature space using two fully connected layers, each followed by a RELU activation function to introduce non-linearity and enhance the capacity of the model for complex representations.

Subsequently, the model employs a linear layer to deliver the SOH estimation, which is represented as a scalar indicating the current health status of the battery. To ensure that the output value falls within the appropriate range of 0–1, a Sigmoid activation function is applied at this stage. Figure 4 provides a visualization of the overall SOH estimation architecture, detailing the progression of information from the convolutional layers, through the self-attention layers, to the fully connected layers. By leveraging this hierarchical feature extraction and learning process, the model successfully converts intricate temporal patterns into a single health status estimation, accurately capturing the SOH trends.

#### Transfer learning

In this study, the potential of transfer learning to enhance SOH estimation for batteries is explored, utilizing generalized features learned from pre-training. This approach proves particularly advantageous for tasks involving diverse charging conditions and intricate battery degradation behaviors, which are prevalent in prediction scenarios. Two datasets are involved herein: dataset #1 employs a four-stage charging protocol (“CC1-CC2-CC3-CV1”) with rates between 3.6C and 6C, and dataset #2 uses a six-stage protocol (“CC1-CC2-CC3-CC4-CC5-CV1”) extending up to 8C. Initially, these datasets are combined; each cell operates under a unique charging rate, leading to varied aging strategies and cycle lifetimes. The differences in charging protocols significantly influence the rate of battery degradation and aging behaviors. The transfer learning framework utilizes the first 70% of the combined datasets as the source domain for training. An additional 10% is used for model validation, while the remaining 20% serves as the target domain for testing. This division ensures that both the source and target domains encompass data from varying operational conditions induced by the different charging protocols. This setup not only tests the ability of the deep learning model to generalize across various charge-discharge rates and aging patterns but also enhances its adaptability to diverse operational scenarios, making it robust in predicting battery health under complex conditions.

During the transfer process, the weights of the pre-trained model were first loaded, providing a solid foundation for the initial parameters of the model at the beginning of transfer learning. These weights serve as a strong starting point by providing general features. Subsequently, data from the test set (target domain) was used for fine-tuning. Since the initial layers of the model (such as convolutional and pooling layers) are used to extract general features, while the higher layers (such as fully connected and multi-head self-attention layers) focus on capturing more specific trends, we chose to freeze the weights of the initial layers during fine-tuning, updating only the parameters of the two multi-head self-attention layers. This strategy ensures that the model retains the general features learned during the pre-training phase while enhancing its sensitivity to new features through targeted fine-tuning of specific layers, thereby effectively improving its performance on new data. Figure 2 provides a detailed explanation of the data sources for the source and target domains in transfer learning, as well as the specific implementation process of transfer learning.

As shown in Table 4, a comparative analysis evaluates the model performance with and without the application of transfer learning. The results demonstrate a significant improvement in SOH estimation accuracy when transfer learning is applied. Specifically, the root-mean-square error (RMSE) decreases from 0.00741 to 0.00109, while R^2^ increases from 0.986 to 0.998, and mean absolute percentage error (MAPE) decreases from 0.00728 to 0.00096. These findings underscore the effectiveness of transfer learning in capturing dataset-specific characteristics and enhancing model accuracy.

**Table 4:** Performance comparison before and after fine-tuning transfer learning

Metric	Before transfer learning	After transfer learning
RMSE (Ah)	0.00609	0.00479
R^2^	0.959	0.977
MAPE (Ah)	0.00481	0.00389

## Results and analysis

This section provides a comprehensive overview of the performance of the proposed model in the task of predicting battery capacity degradation, along with an analysis of various factors influencing the estimation outcomes. Through a thorough evaluation of the performance of the model under different conditions, we not only validate its effectiveness but also delve into the effects of different state of charge (SOC) intervals and sampling intervals on estimation accuracy. In addition, this section compares the computational cost of the model and its differences in accuracy and efficiency with baseline models. The following subsections will examine these key aspects in detail, beginning with the optimal results of the model.

### Results of health estimation

Based on the designed SOH estimation model that integrates a CNN with a multi-head self-attention mechanism, the model effectively extracts local features from battery time-series data while modeling long-range dependencies. By assigning varying weights to different parts of the data, the model captures both long-range dependencies and global context, addressing the limitations of a standalone CNN in capturing global information from battery time-series data. This enables the model to accurately track the degradation trend of battery capacity over the number of cycles and provide precise SOH estimations.

**Figure 5:**
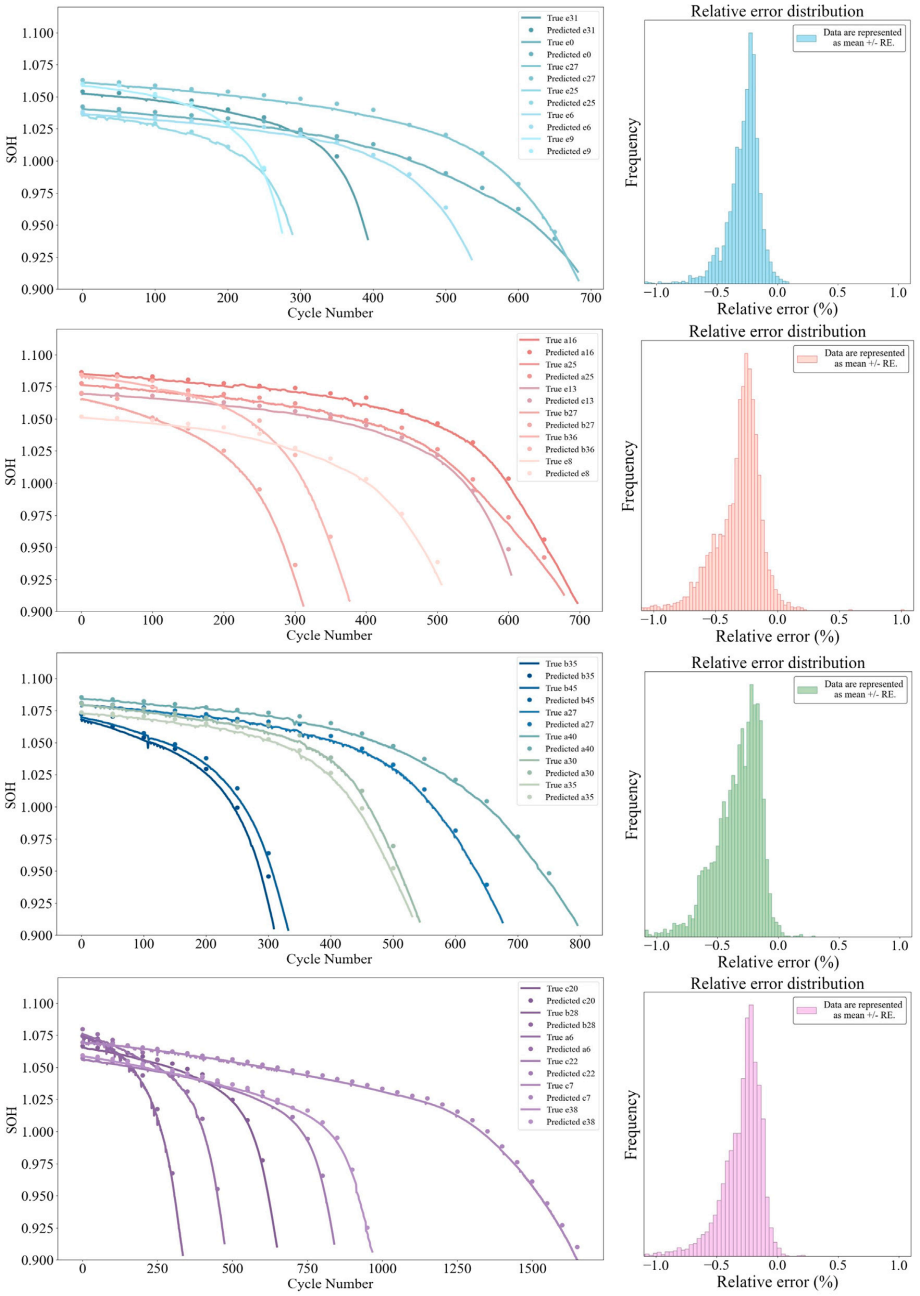
Comparison of predicted and true SOH curves with error distribution for various batteries This figure shows the comparison between the estimated SOH curves and the true SOH values across different cycles (left). The true SOH is represented by solid lines, while the predicted SOH is shown by dotted lines for each battery. The corresponding error distribution histograms (right) illustrate the frequency of estimation errors for each group of batteries, highlighting the performance of the model and the concentration of errors around zero.Data are represented as mean ± RE.

During the experimental process, the raw data underwent preprocessing, where outliers were removed from 222 battery samples, leaving 202 battery datasets for model input. To ensure the training and evaluation processes are representative and accurate, the 202 cleaned battery datasets were split into training, validation, and test sets. Specifically, 142 batteries were used for the training phase to ensure the model learned sufficient feature information, while 21 batteries were allocated to the validation set to help adjust model parameters during training and avoid overfitting. The remaining 39 batteries were used as the test set to assess the final predictive performance of the model. Of the 39 test batteries, 24 were selected, grouped into sets of six, to illustrate the predicted and observed capacity trends over the number of cycles (Figure 5). The histogram in Figure 5 illustrates the distribution of relative errors (REs), which are computed using the formula (predicted value − true value)/true value. In the first few groups of batteries, it is evident that the predicted values closely match the actual capacity trends, demonstrating the ability of the model to effectively capture the degradation process. The error distribution plot further reveals that most estimation errors are concentrated around zero, reflecting the stability and accuracy of the model across different batteries.

**Figure 6:**
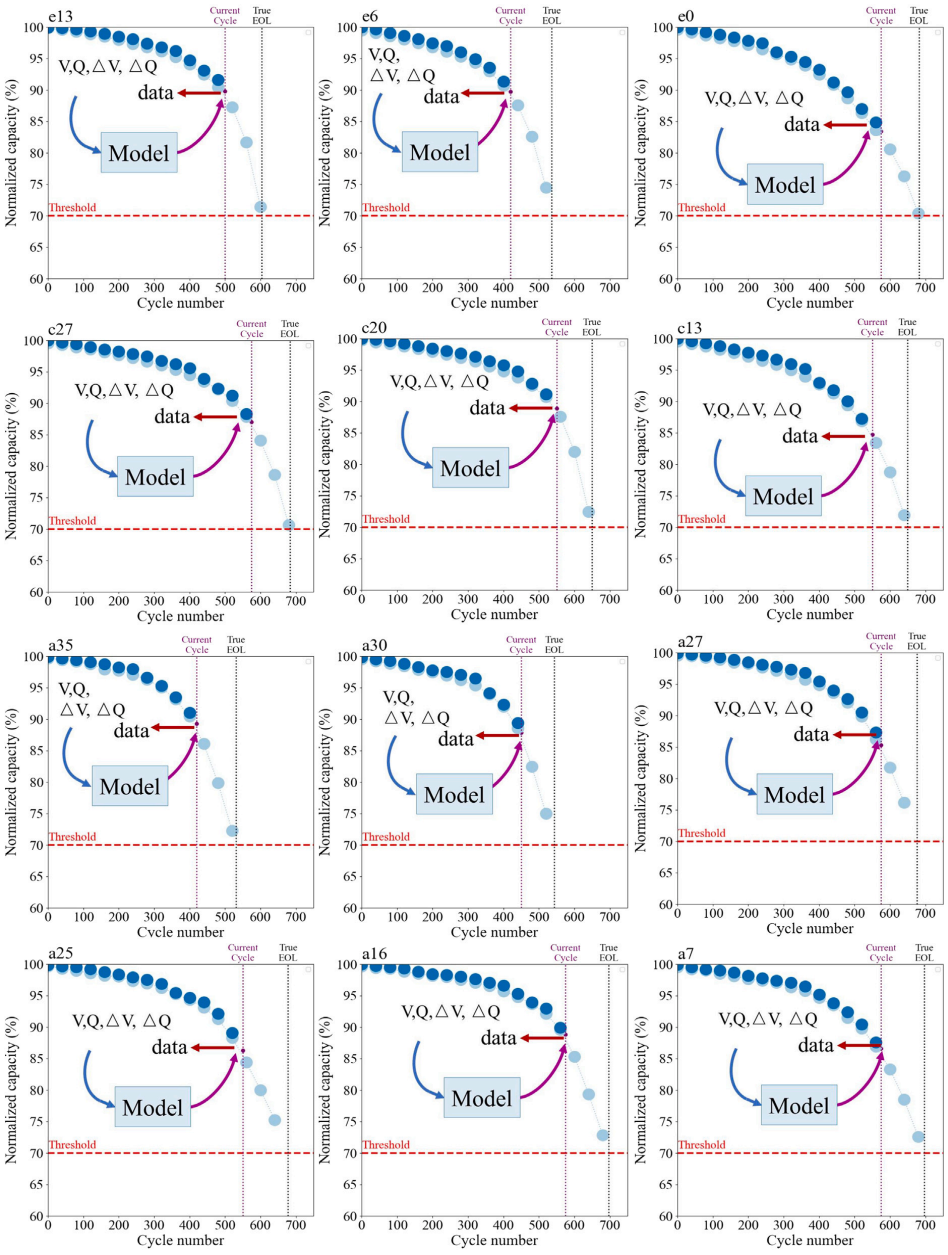
Battery aging trajectory prediction and normalized capacity degradation

Notably, the performance of the model improved significantly after optimization. Metrics including RMSE, R^2^, and MAPE were used to measure the extent of this improvement. Before optimization, the average RMSE across 39 batteries was 0.00609 Ah, with an average R^2^ of 0.959 and an average MAPE of 0.481 Ah. After transfer learning, the performance of the model improved further, with the average RMSE dropping to 0.00479, R^2^ increasing to 0.977, and MAPE reducing to 0.00389. The aging trajectories of selected cells are shown in Figure 6, indicating that the optimized model more accurately captured the capacity degradation trend, substantially enhancing estimation accuracy. Overall, the performance of the model demonstrated considerable improvement after transfer learning. For most batteries, RMSE and MAPE decreased, while R^2^ improved significantly, indicating that the model achieved higher predictive accuracy across most batteries and exhibited strong capability in capturing capacity degradation trends.

### Effect of dataset size

In predicting battery SOH, the division of the dataset plays a critical role in model training and estimation performance, especially for time-series data. This data type requires the model to capture both short-term trends and demonstrate robust generalization capabilities to accurately forecast the future health of batteries. An optimal division of the dataset is crucial, as it influences model learning outcomes and its effectiveness on new, unseen data. A larger training set enables the model to learn a more comprehensive range of features, whereas smaller validation or test sets could limit the assessment of generalization during the tuning process. Thus, selecting appropriate proportions for the training, validation, and test sets is essential to ensure the model effectively learns battery degradation trends and accurately evaluates generalization capabilities during validation and testing. Moreover, the highly nonlinear characteristics and long-term dependencies inherent in battery SOH estimation data can cause model performance to vary significantly with different dataset split ratios. A carefully considered dataset division enhances estimation accuracy and helps prevent errors associated with overfitting or underfitting. This section examines the impact of various dataset split ratios on the SOH estimation model and aims to refine performance for practical applications.

The dataset used in the model consists of 202 battery samples, with 70% allocated to the training set, 10% to the validation set, and 20% to the test set. To ensure the model learns battery degradation trends effectively while maintaining good generalization on future data, we adjusted the proportions of the training, validation, and test sets, designing several dataset division schemes. These schemes were designed based on the specific characteristics of battery SOH estimation tasks, namely the time-series nature and long-term dependencies of the data. The detailed dataset division schemes are shown in Table 5.

**Table 5:** Performance metrics comparison for different dataset split schemes

Scheme	Training set (%)	Validation set (%)	Test set (%)	RMSE (before)	RMSE (after)	R^2^ (before)	R^2^ (after)	MAPE (before)	MAPE (after)
Scheme 1	70	10	20	0.00609	0.00479	0.959	0.977	0.00481	0.00389
Scheme 2	80	10	10	0.00631	0.00404	0.953	0.983	0.00522	0.00323
Scheme 3	75	5	5	0.00745	0.00557	0.939	0.966	0.00584	0.00456
Scheme 4	70	15	15	0.00675	0.00486	0.951	0.976	0.00545	0.00396
Scheme 5	85	5	5	0.00861	0.00738	0.898	0.92	0.00678	0.00574
Scheme 6	65	10	10	0.00698	0.00561	0.937	0.967	0.00552	0.00464

A larger proportion of the training set generally enhances the ability of the model to learn from time-series data. Especially in the context of highly nonlinear battery health states, increasing the size of the training set helps the model capture more complex degradation patterns. Therefore, in the 80%–10%–10% and 85%–5%–10% schemes, the larger training set maximizes data utilization, enabling the model to train on a broader range of data. The validation set plays a key role in monitoring generalization during training, particularly to prevent overfitting. The 70%–15%–15% scheme provides more validation data, allowing for more frequent monitoring of the performance of the model, ensuring stability throughout the training process. The test set is used to evaluate how the model performs on unseen data. A larger test set allows for a more comprehensive assessment of the generalization ability of the model, particularly before deployment, ensuring reliable performance. The 65%–10%–25% split preserves 25% of the data for testing, allowing for a detailed evaluation of how well the model generalizes to unseen data.

To visually compare the effects of different dataset split ratios on model performance, radar charts were employed to present the performance of six key metrics (including RMSE, R^2^, and MAPE) under different schemes. Radar charts allow for the simultaneous display of data across multiple dimensions, facilitating comparisons of multiple results within a single figure. This helps identify the strengths and weaknesses of each scheme across several metrics and reveals the performance trends under different dataset splits.

For visualization, we first computed the average RMSE, R^2^, and MAPE values for each dataset split scheme. To enable an intuitive comparison of these metrics, which are on different scales, we normalized the data before plotting them on the same radar chart. This normalization scaled each value to the [0, 1] range, preventing visualization bias caused by differences in data magnitude and ensuring that each metric was equally represented in the chart. This approach provides a balanced portrayal of the model performance across various dimensions, allowing for a more comprehensive comparison of the different dataset split schemes.

**Figure 7:**
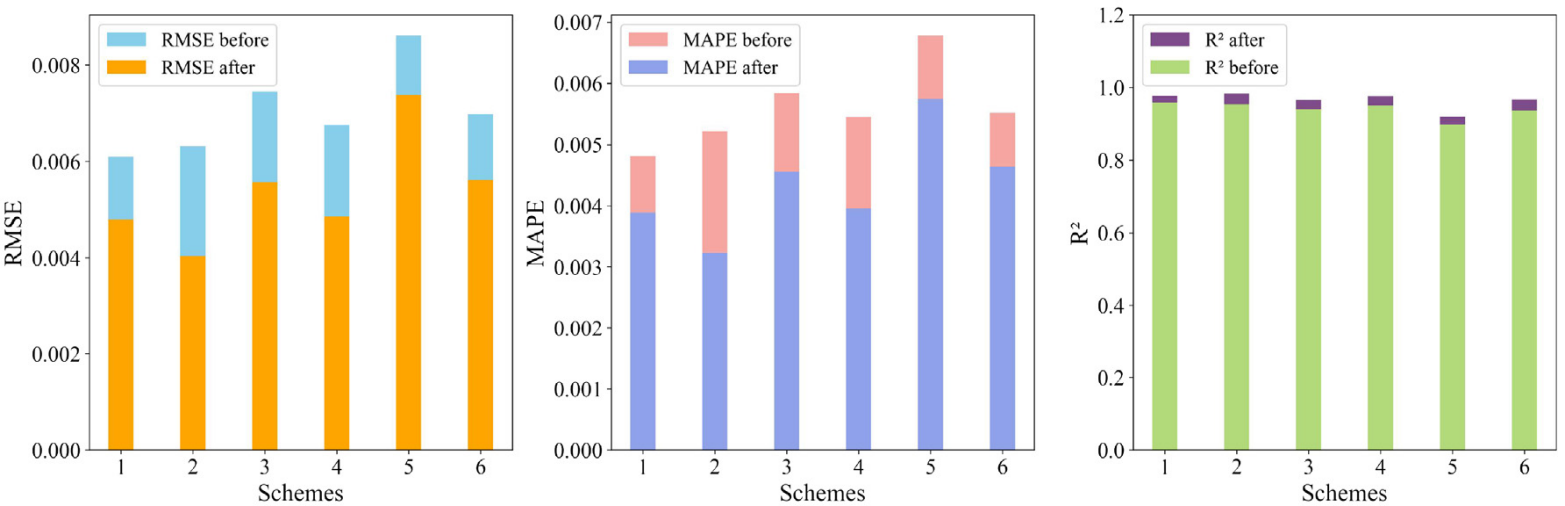
The stacked bar charts of SOH estimation model performance across different dataset split schemes Comparison of RMSE, MAPE, and R^2^ metrics before and after transfer learning across different schemes. The stacked bar charts represent the performance improvements for each metric, with the “before” values displayed in one color and the “after” values stacked on top in a different color. The results demonstrate significant improvements in RMSE and MAPE, while R^2^ values increase, reflecting the enhanced accuracy and robustness of the model after modifications.

As demonstrated in Table 5 and Figure 7, the findings suggest that varying dataset split ratios have a notable influence on the prediction performance of the model. Overall, after optimization, all performance metrics improved, with RMSE and MAPE decreasing notably and R^2^ increasing significantly, suggesting an enhancement of the generalization ability. The model trained with the 70%–10%–20% split (scheme 1) demonstrated well-balanced performance after optimization. Specifically, in scheme 1, RMSE decreased to 0.00479, R^2^ increased to 0.977, and MAPE dropped to 0.00389, indicating that this split effectively balances estimation accuracy and generalization capability. In contrast, while increasing the proportion of the training set (as in scheme 2, with an 80%–10%–10% split) further reduced RMSE to 0.00404 and increased R^2^ to 0.983, the 70%–10%–20% split still provided high estimation accuracy while maintaining a larger test set. The relatively balanced proportions of the validation and test sets ensured the stability of the model and the reliability of the evaluation, making this split advantageous for broader generalization assessments. In addition, in scheme 4, the validation set proportion was increased to 15%, while the test set was reduced to 15%, compared to scheme 1. Despite having the same training set size, the performance of scheme 4 after optimization was slightly inferior. Its RMSE decreased from 0.00675 to 0.00486, R^2^ improved from 0.951 to 0.976, and MAPE decreased from 0.00545 to 0.00396. While performance improved, it was not as remarkable as in scheme 1. The reduction in the test set proportion may have limited the generalization ability of the model on unseen data, preventing a thorough evaluation of its actual performance.

Schemes 3 (75%–5%–20%) and 6 (65%–10%–25%) share some similarities, particularly in their larger test set proportions. The post-optimization RMSE and R^2^ values were similar, with scheme 3 achieving an RMSE of 0.00557 and an R^2^ of 0.966, while scheme 6 had an RMSE of 0.00561 and an R^2^ of 0.967. This suggests that despite the smaller training set size, the larger test set proportion allowed the model to be evaluated on more unseen data, improving its generalization ability and practical application performance. Both schemes also performed similarly in terms of MAPE, with scheme 3 decreasing from 0.00584 to 0.00456 and scheme 6 from 0.00552 to 0.00464, demonstrating good error control. However, due to the smaller training data size, both schemes may struggle in complex scenarios, particularly with certain test samples where the error distribution remained large, indicating the inability of the model to fully capture the complex degradation trends of the battery. These results suggest that although a larger test set improves the evaluation and generalization ability of the model, an appropriate training set proportion is still key to enhancing overall model performance.

It is worth noting that in scheme 5 (85%–5%–10%), although the higher proportion of the training set theoretically facilitates better learning of data features, the optimized RMSE only decreased from 0.00861 to 0.00738, and R^2^ improved from 0.898 to 0.92. Compared to other schemes, the increase in R^2^ was smaller, and MAPE decreased from 0.00678 to 0.00574, with a relatively limited reduction. This may be due to the small proportions of the validation and test sets, causing the model to easily overfit during training. The limited test data were insufficient to thoroughly evaluate the generalization ability of the model, as also reflected in the radar chart in Figure 7. The radar chart shows that while RMSE, MAPE, and R^2^ improved after optimization, the performance of scheme 5 in these metrics was not as strong as in other schemes, particularly in R^2^ and MAPE, where it lagged significantly. The results of this scheme indicate that having too much training data do not significantly enhance the generalization performance of the model, and an appropriate balance between validation and test set proportions is critical for stability.

Overall, the 70%–10%–20% dataset split chosen for this model strikes an effective balance between estimation accuracy and generalization capability. The optimized results show significant reductions in RMSE and MAPE, with R^2^ increasing to 0.977, indicating that the model demonstrates robust predictive performance across a wide range of battery samples. Compared to other dataset split schemes, this ratio effectively avoids overfitting while ensuring good adaptability to unseen data, making it a highly effective strategy for SOH estimation.

### Effect of voltage operating window

In the task of predicting battery SOH, selecting the appropriate SOC range is crucial, as batteries exhibit different capacity degradation characteristics and performance variations across different SOC ranges. Typically, the degradation trend of a battery remains relatively stable in the early and mid-stages of the SOC range, while in certain specific ranges, capacity degradation may accelerate or show more complex nonlinear behavior. Therefore, by choosing an appropriate SOC range, the model can more precisely learn the trends of capacity degradation in the battery, thereby improving estimation accuracy. Narrowing the SOC range allows the model to focus on learning the degradation patterns within a specific range. In a narrower SOC range, the model can capture more detailed features. However, while reducing the SOC range can improve estimation accuracy within that specific range, it also reduces the availability of features in the overall dataset. This may result in insufficient data diversity during training, potentially leading to fluctuations in accuracy or a decrease in the generalization capability of the model. Thus, studying the impact of different SOC ranges on the performance of SOH estimation models can provide important insights for optimizing model performance. By comparing and analyzing the performance of the model across different SOC ranges, it becomes possible to determine in which ranges the model can provide optimal estimation accuracy and how to balance the trade-off between range width and estimation precision.

In this study, to ensure that the input data accurately reflect the degradation trends of the battery, SOC features were extracted when the charging current of the battery reached close to 1 A, voltage reached 3.6 V, and the battery capacity declined to 80% of the nominal capacity. Specifically, the SOC range from 80% to 97% was selected as the main input range for the model, as this range covers the critical stages of capacity degradation, particularly during the early and intermediate stages, allowing for a more comprehensive capture of the global characteristics of capacity changes. This choice enhances the ability of the model to learn the degradation trends of the battery and improves its estimation accuracy.

To further explore the impact of different SOC ranges on the estimation performance of the model before and after transfer learning, we narrowed the SOC range to 80%–95%, 80%–93%, 80%–92%, and 80%–90%. Based on these ranges, battery features were re-extracted, and the model was retrained. During the testing phase, data from 39 battery samples were used to calculate the MAPE value for each battery, which was employed to evaluate the accuracy of the estimations of the model. By combining violin plots, scatterplots, and boxplots, we visually displayed the distribution of estimation errors across different SOC ranges. The violin plot shows the probability density of the MAPE distribution, providing an intuitive view of the overall error trends. While usually symmetric, the plot here is simplified by displaying only the left side. The overlaid scatterplot displays the specific error for each battery, revealing the performance of the model in predicting individual batteries, while the boxplot shows the central tendency of the errors and the presence of outliers, reflecting the distribution of errors and changes in quartiles across different SOC ranges.

**Figure 8:**
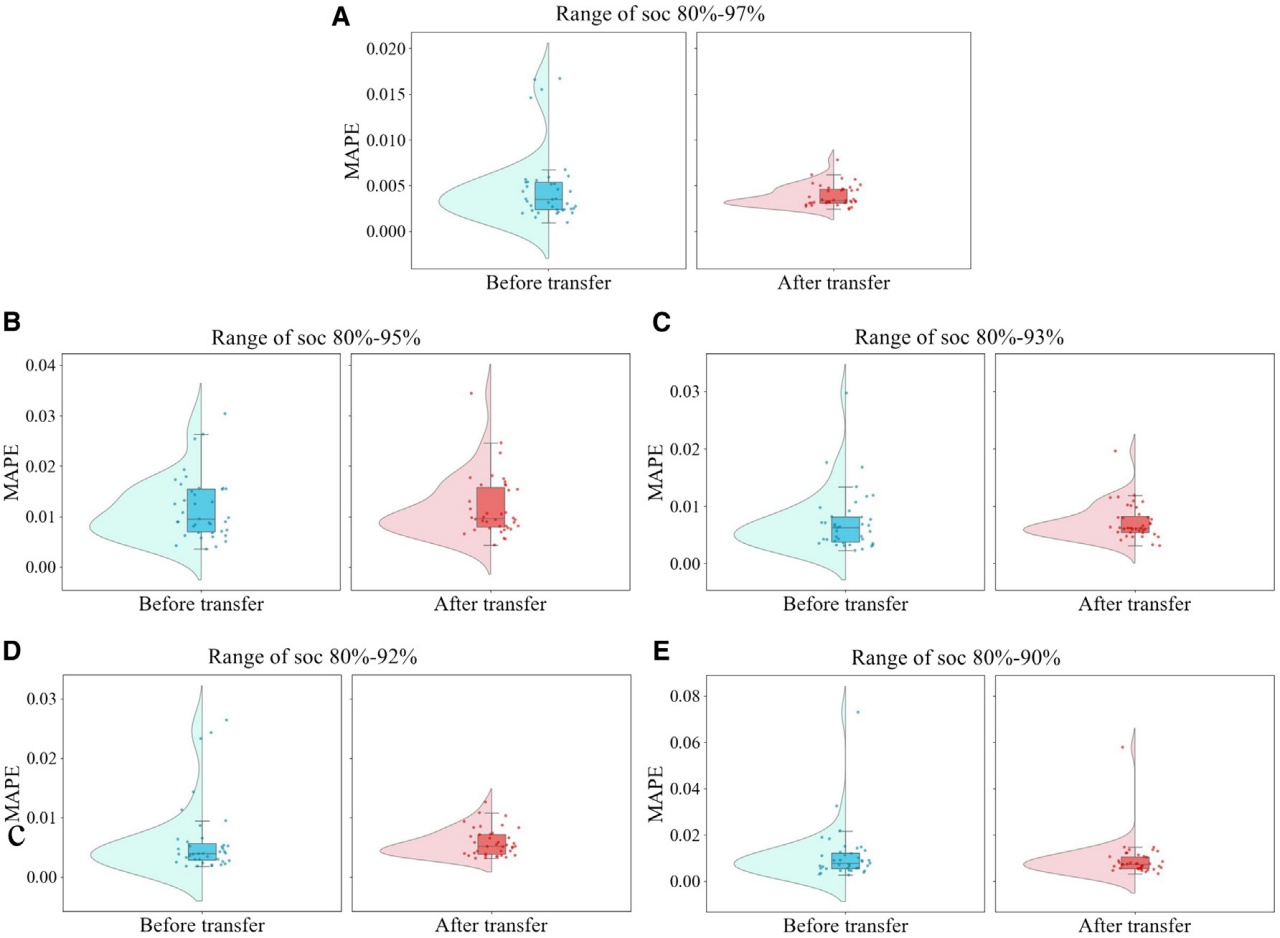
Impact of SOC intervals on estimation diagram This figure presents a comparison of battery health estimation performance before and after applying transfer learning, with a focus on different SOH ranges from 80% to 97%. (A–E) illustrate the MAPE distribution across five SOH ranges. The left side of each panel shows the MAPE before transfer learning, while the right side shows the MAPE after transfer learning.

In comparing different SOC ranges, the SOC range of 80%–97% exhibited the best predictive performance. The violin plot in Figures 5 and 8 shows that after transfer learning, the error distribution significantly narrowed, the long-tail effect diminished, and the quartile range in the boxplot became smaller, with the median decreasing. The scatterplot also indicated that most batteries had smaller estimation errors. As shown in Table 6, RMSE and MAPE dropped to 0.00479 and 0.00389, respectively, while R^2^ increased to 0.977, demonstrating that the ability of the model to learn within this wider SOC range was strong, offering good generalization and stability. In contrast, the SOC range of 80%–95% displayed a slightly wider error distribution, with the scatterplot showing more batteries with larger estimation deviations. RMSE and MAPE increased to 0.0166 and 0.0119, respectively, and R^2^ decreased to 0.691, indicating a decline in the accuracy of the model. Further narrowing to SOC 80%–93% resulted in an even larger error distribution, with the long-tail effect becoming more pronounced. RMSE and MAPE increased to 0.00886 and 0.0073, respectively, and R^2^ dropped to just 0.616, indicating that the ability of the model to capture capacity degradation trends weakened. In the narrowest SOC range of 80%–90%, the long-tail effect in the error distribution was most apparent, with the quartile range in the boxplot expanding significantly. The scatterplot showed an increase in batteries with larger estimation errors. RMSE and MAPE were 0.0122 and 0.00933, respectively, while R^2^ further dropped to 0.404. In summary, a wider SOC range (80%–97%) helped improve the accuracy and generalization capability of the model, whereas its performance gradually deteriorated as the range was narrowed.

**Table 6:** Comparison of model estimation performance across different SOC intervals

Soc interval	RMSE (Ah)	MAPE (Ah)	R2
80%–97%	0.00479	0.00389	0.977
80%–95%	0.0166	0.0119	0.691
80%–93%	0.00886	0.0073	0.616
80%–92%	0.00728	0.00584	0.641
80%–90%	0.0122	0.00933	0.404

As illustrated by the violin plots, boxplots, and scatterplots, the distribution of errors expanded progressively as the SOC range narrowed, with estimation accuracy decreasing significantly in the 80%–90% range. In contrast, the SOC range of 80%–97% provided the best estimation accuracy, with R^2^ of 0.977 and RMSE and MAPE of 0.00479 and 0.00389, respectively. This suggests that selecting a wider SOC range, such as 80%–97%, can effectively enhance the accuracy of the estimations of the model while ensuring robustness in capturing the global characteristics of battery capacity degradation.

### Effect of sampling intervals

**Figure 9:**
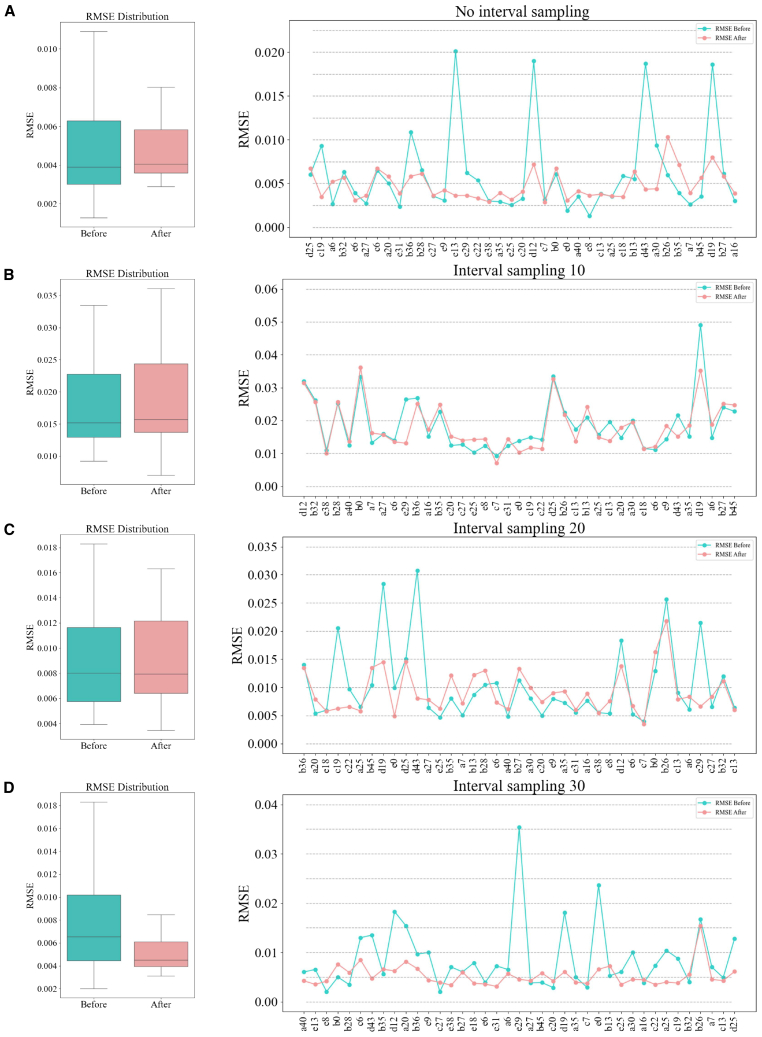
Effect of sampling intervals within fixed soc diagram This figure evaluates RMSE distribution and model performance with varying interval sampling techniques. As shown in (A–D) of the figure. The left column shows boxplots representing RMSE before and after transfer learning under different interval sampling conditions. The right column depicts line graphs that illustrate RMSE trends across different datasets, comparing no interval sampling with sampling intervals of 10, 20, and 30. These visualizations highlight the influence of interval sampling on estimation accuracy and the benefits of transfer learning in improving SOH estimation.

In battery SOH estimation, the selection of the sampling interval is one of the key factors influencing the performance of the model. During the feature extraction process, the size of the sampling interval determines the density of the data, which in turn affects the ability of the model to capture the degradation trends of the battery. Within a fixed SOC range, the sampling interval not only affects the size of the data but also determines the depth of feature extraction. An excessively large sampling interval may lead to the loss of detailed information, while an overly small interval may introduce excessive redundant data, increasing the risk of overfitting. Therefore, selecting an appropriate sampling interval is critical to ensuring the predictive performance of the model and optimizing the effectiveness of feature extraction. In this study, we conducted experiments to evaluate the impact of different sampling intervals within a fixed SOC range (80%–97%) on the performance of the model. The sampling process was conducted using voltage and charge capacity as features. A baseline sampling interval of 10 was set in the code, but to comprehensively assess the impact of sampling intervals, we also tested intervals of 20 and 30. For each sampling interval, the model was retrained using the same training data and tested on a dataset of 39 batteries. This experimental design allowed us to systematically analyze the effects of different sampling intervals on feature extraction and the predictive performance of the model. In the original model, without any interval sampling, it demonstrated high accuracy. After transfer learning, RMSE dropped from 0.00609 to 0.00479, R^2^ improved from 0.959 to 0.977, and MAPE decreased from 0.00481 to 0.00389. The curve plot (Figure 9) shows that after transfer learning, the error distribution became more compact, particularly for test samples with larger errors, which saw a significant reduction. The boxplot reflected the same trend, with the interquartile range narrowing and the median decreasing, indicating that the model was able to accurately capture SOH changes without interval sampling, and transfer learning significantly improved the stability and predictive accuracy.

For a sampling interval of 10, the curve plot shows that large errors in certain battery samples remained unaddressed, with significant fluctuations in the curve, indicating that the error distribution remained widespread. The boxplot similarly showed a more dispersed error distribution, with a relatively wide interquartile range and some samples exhibiting large deviations. This suggests that a smaller sampling interval did not allow the model to fully learn the degradation trends of the batteries, leading to weaker overall performance. In contrast, the sampling interval of 20 showed significant performance improvements, with RMSE dropping from 0.00868 to 0.00462, R^2^ improving from 0.899 to 0.967, and MAPE decreasing from 0.00711 to 0.00364. The curve plot shows that after transfer learning, the error distribution narrowed significantly, with reduced fluctuations, and errors in most test samples were substantially reduced. The boxplot also confirmed this result, with a significantly reduced interquartile range, a lower median, and fewer outliers, demonstrating that the model was better able to capture the subtle changes in battery degradation. Under this interval, both the estimation accuracy and stability of the model were greatly enhanced. However, with a sampling interval of 30, the performance of the model slightly declined, though it remained at a high level. RMSE dropped from 0.00877 to 0.00528, R^2^ improved from 0.894 to 0.966, and MAPE decreased from 0.00727 to 0.00423. The curve plot shows some improvement after transfer learning, but not as significant as with an interval of 20, and fluctuations in certain battery samples remained. The boxplot reflects that although the error distribution remained relatively concentrated, the interquartile range was slightly wider compared to the sampling interval of 20. This indicates that as the sampling interval increased further, the performance and predictive accuracy of the model began to decline, especially in the performance of certain battery samples.

In summary, comparing the effects of different sampling intervals reveals that the sampling interval significantly impacts the predictive performance. An interval of 20 produced the best results, with RMSE and MAPE reaching their lowest values and R^2^ closely matching the performance of the original model. This indicates that an optimal sampling interval enhances the model accuracy. From the curve trends and boxplot distributions, it can be observed that a sampling interval of 20 resulted in smoother curves, fewer error peaks, a clear concentration of errors, and a narrow interquartile range. In contrast, an interval of 10 was too small to capture global information, leading to large error fluctuations, as shown by the more dispersed error distribution in the boxplot and prominent errors in certain battery samples. While an interval of 30 showed improvements for some batteries, the larger interval caused a loss of detail, leading to decreased performance and less ideal error concentration compared to the interval of 20.

Therefore, in this experiment, an interval of 20 was the most suitable choice for sampling. A proper sampling interval not only helps the model better capture the degradation trends of the battery, thereby improving estimation accuracy, but also suppresses error fluctuations and maintains the stability of the estimation results.

### Performance comparison and ablation analysis

CNN and LSTM are used as baseline models to evaluate the performance of the proposed hybrid fusion model in terms of accuracy, robustness, and generalization. CNN and LSTM are widely applied in SOH estimation due to their established relevance and effectiveness in this field. For comparison, the CNN model consists of three convolutional layers, each followed by a max-pooling layer, while the LSTM model includes a single LSTM layer and a fully connected layer.

To further analyze the contribution of individual components in the proposed model, ablation studies were conducted. These experiments evaluated the performance of the model using only the CNN module or only the self-attention layer, aiming to assess the importance of each component. By removing key modules, the significance and advantages of the proposed model were further verified. To ensure fairness and relevance, all models were evaluated under identical experimental conditions. Both CNN and LSTM used the same dataset as the proposed model, and the dataset underwent consistent preprocessing to ensure data uniformity. Specifically, the dataset was split into 70% training, 10% validation, and 20% testing subsets. In addition, hyperparameters such as learning rate, batch size, and training epochs were kept consistent across models.

**Figure 10:**
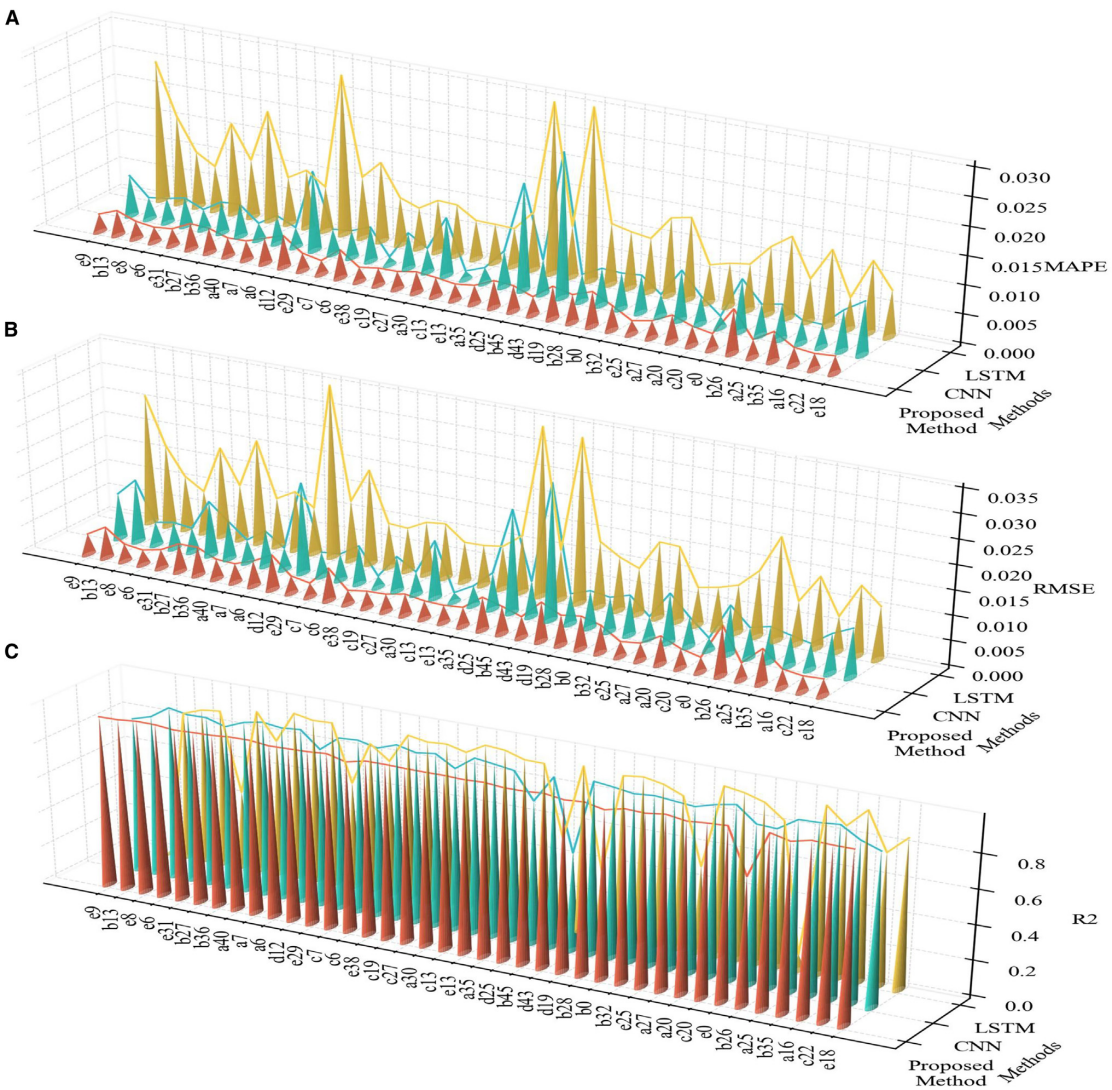
Comparison of model performance metrics across different methods The figure presents the performance comparison of different models (LSTM, CNN, and the proposed method) for battery SOH prediction, covering three metrics: (A) RMSE, (B) MAPE, and (C) R^2^. The proposed method demonstrates significant superiority in minimizing errors and achieving higher prediction accuracy.

Table 7 provides a clear comparison of the performance of the proposed hybrid model (CNN + self-attention) and the baseline models (CNN, attention, and LSTM) in terms of RMSE, R^2^, and MAPE. The results indicate that the fusion of multi-neural networks achieves superior performance compared to the baseline models. These findings highlight the importance of the CNN module in capturing local features and the critical role of the self-attention module in representing global dependencies. The superior performance of the hybrid model is attributed to the synergistic integration of CNN and self-attention modules, underscoring the rationale and effectiveness of the proposed architecture. Figure 10 further visualizes these performance trends, with the hybrid model demonstrating smoother result curves across all samples. This indicates its superior accuracy, robustness, and generalization ability. Figure 10 reveals that the hybrid model consistently achieves lower RMSE and MAPE values across all battery samples, and its R^2^ remains close to 0.98, reflecting its ability to accurately capture battery performance trends over time. In contrast, the CNN and LSTM models exhibit higher RMSE and MAPE fluctuations across some samples, leading to greater prediction errors. These experimental results highlight the limitations of baseline models in handling complex battery SOH prediction tasks, while the hybrid model leverages CNN for local feature extraction and the self-attention layer for global dependency modeling, significantly improving prediction accuracy and stability.

**Table 7:** Ablation and comparison study on CNN and self-attention components

Model	RMSE (Ah)	R^2^	MAPE (Ah)
Fusion model (CNN+ self-attention)	0.00479	0.977	0.00389
CNN	0.00696	0.934	0.00555
Self-attention	0.02000	0.643	0.0155
LSTM	0.01400	0.800	0.0121

### Conclusion

This study systematically analyzed the estimation of the SOH of LIBs using a hybrid deep learning model, combining CNNs and a multi-head self-attention mechanism. The proposed model effectively addressed the challenge of accurately predicting SOH under variable fast-charging conditions, especially when limited segment data from the SOC are available. The results indicate that the model significantly improves SOH prediction accuracy during critical battery operation phases, particularly in the SOC range of 80%–97%, where battery degradation is most pronounced. The effectiveness of the proposed approach was evaluated using two datasets, resulting in exceptional performance with an average MAPE of 0.389%, an RMSE of 0.479%, and a R^2^ of 97.7%. These findings not only enrich the existing theories in battery health diagnostics but also provide a robust framework for practical applications in battery management systems, especially for fast-charging scenarios.

Despite these promising results, the study faces certain limitations, including the dependency on a specific range of SOC data and the challenge of incorporating a wider variety of battery chemistries or aging behaviors. These issues offer avenues for future research, such as extending the applicability of the model to other SOC ranges and battery types, or enhancing its performance by incorporating additional environmental variables. Future studies could also explore the integration of real-time data collection techniques and model refinement to further enhance predictive capabilities. Overall, the research presented in this paper provides valuable theoretical support for advancing battery health management systems and contributes to the further development of the field of battery diagnostics and maintenance.

## Discussion

The proposed model represents a significant advancement in SOH estimation for lithium-ion batteries under fast-charging conditions, addressing key challenges in real-time battery diagnostics. Unlike conventional methods that rely on extensive feature engineering and handcrafted models, this deep learning-based approach achieves high predictive accuracy while requiring only voltage and charge capacity inputs. The integration of CNNs and multi-head self-attention mechanisms enables both local feature extraction and global dependency analysis, improving the robustness of SOH estimation. A key contribution of this study is the ability to operate within a limited SOC range (80%–97%), capturing degradation trends in the most critical phase of battery aging. This targeted approach enhances the precision compared to traditional SOH estimation techniques that attempt to generalize across the entire SOC spectrum. In addition, the use of fast-charging datasets (3.6C–8C charge, 4C discharge) provides insights into battery behavior under extreme operational conditions, making this framework particularly relevant for applications in EVs, grid storage, and portable electronics. Beyond its practical implications, this research also contributes to the broader field of battery diagnostics and machine learning by demonstrating how deep learning architectures can be optimized for resource-efficient, real-time SOH prediction. The findings reinforce the potential of hybrid neural networks in capturing complex battery degradation dynamics while reducing reliance on computationally expensive physics-based models. Future research can explore adaptive learning techniques that allow the model to update dynamically as new battery data becomes available, further enhancing its real-world applicability. By bridging the gap between data-driven SOH estimation and real-world implementation, this study lays the groundwork for next-generation battery management system (BMS) that prioritize efficiency, accuracy, and scalability.

### Limitations of the study

Despite the strong performance of the proposed hybrid deep learning model in estimating battery SOH under fast-charging conditions, several aspects require further investigation. Model interpretability remains a key challenge, necessitating deeper analysis to enhance the transparency and reliability of deep learning-based predictions. In addition, the dataset primarily reflects specific charging protocols, which may not fully capture the variability of real-world operational conditions. The reliance on high-quality labeled data presents challenges in data-limited environments, potentially restricting its broader applicability. Furthermore, its computational complexity may impede real-time deployment in BMS. Future research should focus on expanding the applicability across diverse battery chemistries, charging conditions, and environmental factors, while optimizing computational efficiency to facilitate practical implementation.

## Resource availability

### Lead contact

Requests for further information and resources should be directed to and will be fulfilled by the lead contact, Jingyuan Zhao (jyzhao@ucdavis.edu).

### Materials availability

This study did not generate new materials.

### Data and code availability

• The datasets used in this study are publicly available. The dataset can be accessed at the following URL: https://data.matr.io/1.
• The original code is available from the lead contact upon a reasonable request.
• Any additional information in this paper is available from the lead contact upon request

## Acknowledgments

This research was funded by Shenzhen Science and Technology Program, grant number 29853M-KCJ-2023-002-13, and Independent Innovation Projects of the Hubei Longzhong Laboratory, grant number 2022ZZ-24.

## Author contributions

J.Z., conceptualization, methodology, formal analysis, software, writing – original draft, supervision. D.L., data curation, software, investigation, visualization, writing – original draft. Y.L., formal analysis, methodology, validation. D.S., formal analysis, resources, funding acquisition, investigation. J.N., supervision, resources, funding acquisition. A.F.B., conceptualization, writing – review & editing, supervision.

## Declaration of interests

The authors declare no competing interests.

## STAR★Methods

### Key resources table

**Table:** 

REAGENT or RESOURCE	SOURCE	IDENTIFIER
**Deposited data**		

LFP/graphite	Open-source datasets^69^^,^^70^	https://doi.org/10.1038/s41586-020-1994-5

**Software and algorithms**		

Python 3.9	Python Software Foundation	https://www.python.org

### Method details

#### Data preprocessing

Data preprocessing involves several key steps, including raw data extraction, feature normalization, feature construction, and dataset segmentation. Invalid battery data, such as those not reaching 80% discharge capacity and noisy channel data, are removed. Key features of the battery cycling data, such as voltage, charge capacity, and discharge capacity, are normalized. A sliding window approach is applied to generate input features, ensuring that the model captures time-series dependencies. Finally, the dataset is split into training (70%), validation (10%), and test (20%) sets to optimize model generalization and performance.

#### Feature engineering

Feature engineering focuses on extracting and constructing key features that represent battery SOH degradation trends. Voltage and charge capacity are selected as primary input features, while discharge capacity serves as an auxiliary target to enhance the ability of the model to identify degradation patterns. To quantify performance decline over cycles, the voltage curve of each cycle is compared to the voltage curve of the 10th cycle, and voltage and capacity differences are computed as derived features. Linear interpolation is applied to standardize all features to 100 time steps, ensuring data continuity and consistency before normalization and input into the model.

#### Model selection

A hybrid deep learning model combining CNNs and Multi-Head Self-Attention is proposed for SOH estimation. The CNN layers extract local time-series features, while the self-attention mechanism captures long-term dependencies, enhancing prediction accuracy. The architecture consists of three convolutional blocks for local feature extraction, followed by max-pooling layers to reduce dimensionality while preserving critical information. After passing through fully connected layers, the multi-head self-attention mechanism models global dependencies, ultimately producing SOH predictions.

#### Model training

The training process is conducted using a batch size of 512 and an initial learning rate of 8e-4. An early stopping strategy (patience=1600) is implemented to prevent overfitting. In the transfer learning stage, the model is pre-trained on the source domain dataset (70% of the data) before fine-tuning on the target domain dataset (20% of the data). The lower convolutional layers remain frozen, while the higher self-attention layers are fine-tuned, improving adaptability to new data while reducing computational cost.

#### Model evaluation

Model evaluation is performed using multiple metrics, including RMSE, MAPE, and the R^2^. Before applying transfer learning, the model achieves an RMSE of 0.00609 Ah, MAPE of 0.00481 Ah, and R^2^ of 0.959. After transfer learning optimization, RMSE improves to 0.00479 Ah, MAPE decreases to 0.00389 Ah, and R^2^ increases to 0.977, demonstrating enhanced generalization and predictive performance. Additionally, ablation studies verify the contribution of each model component, showing that the hybrid CNN-Self-Attention model significantly outperforms standalone CNN or LSTM-based models for SOH estimation.

### Quantification and statistical analysis

The study utilizes common statistical metrics such as RMSE, R^2^, and MAPE to evaluate the accuracy of the battery SOH predictions. These metrics are applied across different experimental conditions, including transfer learning and comparison with baseline models. The deep learning models were implemented using Python 3.9, which was employed for data preprocessing, model training, and performance evaluation.

## References

[1] Dunn B., Kamath H., Tarascon J.M. (2011). Electrical Energy Storage for the Grid: A Battery of Choices. Science.

[2] Schmuch R., Wagner R., Hörpel G., Placke T., Winter M. (2018). Performance and Cost of Materials for Lithium-Based Rechargeable Automotive Batteries. Nat. Energy.

[3] Kelly F.J., Zhu T. (2016). Transport Solutions for Cleaner Air. Science.

[4] Burke A.F., Zhao J., Fulton L.M. (2024). Projections of the Costs of Light-Duty Battery-Electric and Fuel Cell Vehicles (2020–2040) and Related Economic Issues. Res. Transport. Econ..

[5] Burke A.F., Zhao J., Miller M.R., Sinha A., Fulton L.M. (2023). Projections of the Costs of Medium- and Heavy-Duty Battery-Electric and Fuel Cell Vehicles (2020-2040) and Related Economic Issues. Energy for Sustainable Development.

[6] Liu K., Liu Y., Lin D., Pei A., Cui Y. (2018). Materials for Lithium-Ion Battery Safety. Sci. Adv..

[7] Zhao J., Nan J., Wang J., Ling H., Lian Y., Burke A. (2022). 2022 IEEE Vehicle Power and Propulsion Conference (VPPC).

[8] Palacín M.R., de Guibert A. (2016). Why Do Batteries Fail?. Science.

[9] Zhao J., Ling H., Liu J., Wang J., Burke A.F., Lian Y. (2023). Machine Learning for Predicting Battery Capacity for Electric Vehicles. ETransportation.

[10] Zhao J., Ling H., Wang J., Burke A.F., Lian Y. (2022). Data-Driven Prediction of Battery Failure for Electric Vehicles. iScience.

[11] Wang Z., Shi D., Zhao J., Chu Z., Guo D., Eze C., Qu X., Lian Y., Burke A.F. (2024). Battery Health Diagnostics: Bridging the Gap Between Academia and Industry. ETransportation.

[12] Lin C., Tang A., Mu H., Wang W., Wang C. (2015). Aging Mechanisms of Electrode Materials in Lithium-Ion Batteries for Electric Vehicles. J. Chem..

[13] Zhang S., Zhao K., Zhu T., Li J. (2017). Electrochemomechanical Degradation of High-Capacity Battery Electrode Materials. Prog. Mater. Sci..

[14] Waag W., Käbitz S., Sauer D.U. (2013). Experimental Investigation of the Lithium-Ion Battery Impedance Characteristic at Various Conditions and Aging States and Its Influence on the Application. Appl. Energy.

[15] Wang Y., Xiang H., Soo Y.Y., Fan X. (2025). Aging Mechanisms, Prognostics and Management for Lithium-Ion Batteries: Recent Advances. Renew. Sustain. Energy Rev..

[16] LeCun Y., Bengio Y., Hinton G. (2015). Deep Learning. Nature.

[17] Zhao J., Qu X., Wu Y., Fowler M., Burke A.F. (2024). Artificial Intelligence-Driven Real-World Battery Diagnostics. Energy and AI.

[18] Li H., Xie X., Zhang X., Burke A.F., Zhao J. (2025). Battery state estimation for electric vehicles: Translating AI innovations into real-world solutions. J. Energy Storage.

[19] Ng M.F., Zhao J., Yan Q., Conduit G.J., Seh Z.W. (2020). Predicting the State of Charge and Health of Batteries Using Data-Driven Machine Learning. Nat. Mach. Intell..

[20] Zhao J., Feng X., Pang Q., Wang J., Lian Y., Ouyang M., Burke A.F. (2023). Battery Prognostics and Health Management from a Machine Learning Perspective. J. Power Sources.

[21] Roman D., Saxena S., Robu V., Pecht M., Flynn D. (2021). Machine Learning Pipeline for Battery State-of-Health Estimation. Nat. Mach. Intell..

[22] Zhao J., Feng X., Tran M.K., Fowler M., Ouyang M., Burke A.F. (2024). Battery Safety: Fault Diagnosis from Laboratory to Real World. J. Power Sources.

[23] Tian Y., Lin C., Li H., Du J., Xiong R. (2021). Detecting Undesired Lithium Plating on Anodes for Lithium-Ion Batteries – A Review on the In-Situ Methods. Appl. Energy.

[24] Li J., Landers R.G., Park J. (2020). A Comprehensive Single-Particle-Degradation Model for Battery State-of-Health Estimation. J. Power Sources.

[25] von Kolzenberg L., Latz A., Horstmann B. (2020). Solid–Electrolyte Interphase During Battery Cycling: Theory of Growth Regimes. ChemSusChem.

[26] Liu W., Liu P., Mitlin D. (2020). Review of Emerging Concepts in SEI Analysis and Artificial SEI Membranes for Lithium, Sodium, and Potassium Metal Battery Anodes. Adv. Energy Mater..

[27] Zhao J., Burke A.F. (2023). Battery Prognostics and Health Management for Electric Vehicles Under Industry 4.0. J. Energy Chem..

[28] Thelen A., Lui Y.H., Shen S., Laflamme S., Hu S., Ye H., Hu C. (2022). Integrating Physics-Based Modeling and Machine Learning for Degradation Diagnostics of Lithium-Ion Batteries. Energy Storage Mater..

[29] Yang S., Zhang C., Jiang J., Zhang W., Zhang L., Wang Y. (2021). Review on State-of-Health of Lithium-Ion Batteries: Characterizations, Estimations and Applications. J. Clean. Prod..

[30] Lucu M., Martinez-Laserna E., Gandiaga I., Liu K., Camblong H., Widanage W.D., Marco J. (2020). Data-Driven Nonparametric Li-Ion Battery Ageing Model Aiming at Learning from Real Operation Data-Part B: Cycling Operation. J. Energy Storage.

[31] Qu X., Shi D., Zhao J., Tran M.K., Wang Z., Fowler M., Lian Y., Burke A.F. (2024). Insights and Reviews on Battery Lifetime Prediction from Research to Practice. J. Energy Chem..

[32] Hu X., Xu L., Lin X., Pecht M. (2020). Battery Lifetime Prognostics. Joule.

[33] Zhao J., Burke A.F. (2022). Electric Vehicle Batteries: Status and Perspectives of Data-Driven Diagnosis and Prognosis. Batteries.

[34] Xiong R., Sun Y., Wang C., Tian J., Chen X., Li H., Zhang Q. (2023). A Data-Driven Method for Extracting Aging Features to Accurately Predict the Battery Health. Energy Storage Mater..

[35] Zhao J., Feng X., Pang Q., Fowler M., Lian Y., Ouyang M., Burke A.F. (2024). Battery Safety: Machine Learning-Based Prognostics. Prog. Energy Combust. Sci..

[36] Fu S., Tao S., Fan H., He K., Liu X., Tao Y., Zuo J., Zhang X., Wang Y., Sun Y. (2024). Data-Driven Capacity Estimation for Lithium-Ion Batteries with Feature Matching Based Transfer Learning Method. Appl. Energy.

[37] Wei J., Dong G., Chen Z. (2018). Remaining Useful Life Prediction and State of Health Diagnosis for Lithium-Ion Batteries Using Particle Filter and Support Vector Regression. IEEE Trans. Ind. Electron..

[38] Li Y., Zou C., Berecibar M., Nanini-Maury E., Chan J.C.W., Van den Bossche P., Van Mierlo J., Omar N. (2018). Random Forest Regression for Online Capacity Estimation of Lithium-Ion Batteries. Appl. Energy.

[39] Su X., Sun B., Wang J., Zhang W., Ma S., He X., Ruan H. (2022). Fast Capacity Estimation for Lithium-Ion Battery Based on Online Identification of Low-Frequency Electrochemical Impedance Spectroscopy and Gaussian Process Regression. Appl. Energy.

[40] Li A.G., West A.C., Preindl M. (2022). Towards Unified Machine Learning Characterization of Lithium-Ion Battery Degradation Across Multiple Levels: A Critical Review. Appl. Energy.

[41] Li S., Fang H., Shi B. (2021). Remaining Useful Life Estimation of Lithium-Ion Battery Based on Interacting Multiple Model Particle Filter and Support Vector Regression. Reliab. Eng. Syst. Saf..

[42] Zhao J., Wang Z. (2024). Specialized Convolutional Transformer Networks for Estimating Battery Health via Transfer Learning. Energy Storage Mater..

[43] Fan Y., Xiao F., Li C., Yang G., Tang X. (2020). A Novel Deep Learning Framework for State of Health Estimation of Lithium-Ion Battery. J. Energy Storage.

[44] Zhao J., Han X., Ouyang M., Burke A.F. (2023). Specialized Deep Neural Networks for Battery Health Prognostics: Opportunities and Challenges. J. Energy Chem..

[45] Khaleghi S., Karimi D., Beheshti S.H., Hosen M.S., Behi H., Berecibar M., Van Mierlo J. (2021). Online Health Diagnosis of Lithium-Ion Batteries Based on Nonlinear Autoregressive Neural Network. Appl. Energy.

[46] Zhao J., Feng X., Wang J., Lian Y., Ouyang M., Burke A.F. (2023). Battery Fault Diagnosis and Failure Prognosis for Electric Vehicles Using Spatio-Temporal Transformer Networks. Appl. Energy.

[47] Yang N., Song Z., Hofmann H., Sun J. (2022). Robust State of Health Estimation of Lithium-Ion Batteries Using Convolutional Neural Network and Random Forest. J. Energy Storage.

[48] Ardeshiri R.R., Liu M., Ma C. (2022). Multivariate Stacked Bidirectional Long Short Term Memory for Lithium-Ion Battery Health Management. Reliab. Eng. Syst. Saf..

[49] Yang H., Hong J., Liang F., Xu X. (2023). Machine Learning-Based State of Health Prediction for Battery Systems in Real-World Electric Vehicles. J. Energy Storage.

[50] Tian J., Xiong R., Shen W., Lu J., Sun F. (2022). Flexible Battery State of Health and State of Charge Estimation Using Partial Charging Data and Deep Learning. Energy Storage Mater..

[51] Tang A., Xu Y., Hu Y., Tian J., Nie Y., Yan F., Tan Y., Yu Q. (2024). Battery State of Health Estimation Under Dynamic Operations with Physics-Driven Deep Learning. Appl. Energy.

[52] Lu J., Xiong R., Tian J., Wang C., Sun F. (2023). Deep Learning to Estimate Lithium-Ion Battery State of Health Without Additional Degradation Experiments. Nat. Commun..

[53] Zhao J., Qu X., Han X., Wu Y., Burke A.F. (2025). Cross-Material Battery Capacity Estimation Using Hybrid-Model Fusion Transfer Learning. J. Power Sources.

[54] Shu X., Shen J., Li G., Zhang Y., Chen Z., Liu Y. (2021). A Flexible State-of-Health Prediction Scheme for Lithium-Ion Battery Packs with Long Short-Term Memory Network and Transfer Learning. IEEE Trans. Transp. Electrific..

[55] Che Y., Deng Z., Lin X., Hu L., Hu X. (2021). Predictive Battery Health Management with Transfer Learning and Online Model Correction. IEEE Trans. Veh. Technol..

[56] Zhang J., Mao L., Liu Z., Yu K., Hu Z. (2025). A Bayesian Transfer Learning Framework for Assessing Health Status of Lithium-Ion Batteries Considering Individual Battery Operating States. Appl. Energy.

[57] Ji S., Zhang Z., Stein H.S., Zhu J. (2025). Flexible Health Prognosis of Battery Nonlinear Aging Using Temporal Transfer Learning. Appl. Energy.

[58] Lv Z., Zhao J. (2025). Resource-Efficient Artificial Intelligence for Battery Capacity Estimation Using Convolutional FlashAttention Fusion Networks. eTransportation.

[59] Xu Q., Wu M., Khoo E., Chen Z., Li X. (2023). A Hybrid Ensemble Deep Learning Approach for Early Prediction of Battery Remaining Useful Life. IEEE/CAA J. Autom. Sinica.

[60] Chen B., Liu Y., Xiao B. (2024). A Novel Hybrid Neural Network-Based SOH and RUL Estimation Method for Lithium-Ion Batteries. J. Energy Storage.

[61] Wang X., Dai K., Hu M., Ni N. (2024). Lithium-Ion Battery Health State and Remaining Useful Life Prediction Based on Hybrid Model MFE-GRU-TCA. J. Energy Storage.

[62] (2024). A Hybrid Approach to Predict Battery Health Combined with Attention-Based Transformer and Online Correction. J. Energy Storage.

[63] Lyu Z., Wang G., Gao R. (2021). Li-Ion Battery Prognostic and Health Management Through an Indirect Hybrid Model. J. Energy Storage.

[64] Wen S., Lin N., Huang S., Li X., Wang Z., Zhang Z. (2024). Lithium Battery State of Health Estimation Using Real-World Vehicle Data and an Interpretable Hybrid Framework. J. Energy Storage.

[65] Soo Y.Y., Wang Y., Xiang H., Chen Z. (2024). Machine Learning-Based Battery Pack Health Prediction Using Real-World Data. Energy.

[66] Chae S.G., Bae S.J., Oh K.Y. (2025). State-of-Health Estimation and Remaining Useful Life Prediction of Lithium-Ion Batteries Using DnCNN-CNN. J. Energy Storage.

[67] Zhao J., Wang Z., Wu Y., Burke A.F. (2025). Predictive Pretrained Transformer (PPT) for Real-Time Battery Health Diagnostics. Appl. Energy.

[68] Zhao J., Han X., Wu Y., Wang Z., Burke A.F. (2024). Opportunities and Challenges in Transformer Neural Networks for Battery State Estimation: Charge, Health, Lifetime, and Safety. J. Energy Chem..

[69] Severson K.A., Attia P.M., Jin N., Perkins N., Jiang B., Yang Z., Chen M.H., Aykol M., Herring P.K., Fraggedakis D. (2019). Data-Driven Prediction of Battery Cycle Life Before Capacity Degradation. Nat. Energy.

[70] Attia P.M., Grover A., Jin N., Severson K.A., Markov T.M., Liao Y.H., Chen M.H., Cheong B., Perkins N., Yang Z. (2020). Closed-Loop Optimization of Fast-Charging Protocols for Batteries with Machine Learning. Nature.

